# Supplementary figures and images for: Is prevalence of e-cigarette and nicotine replacement therapy use among smokers associated with average cigarette consumption in England? A time-series analysis
Source: BMJ Open. 2018 Jun 19;8(6):e016046. doi: 10.1136/bmjopen-2017-016046 (PMC6020958; doi:10.1136/bmjopen-2017-016046)

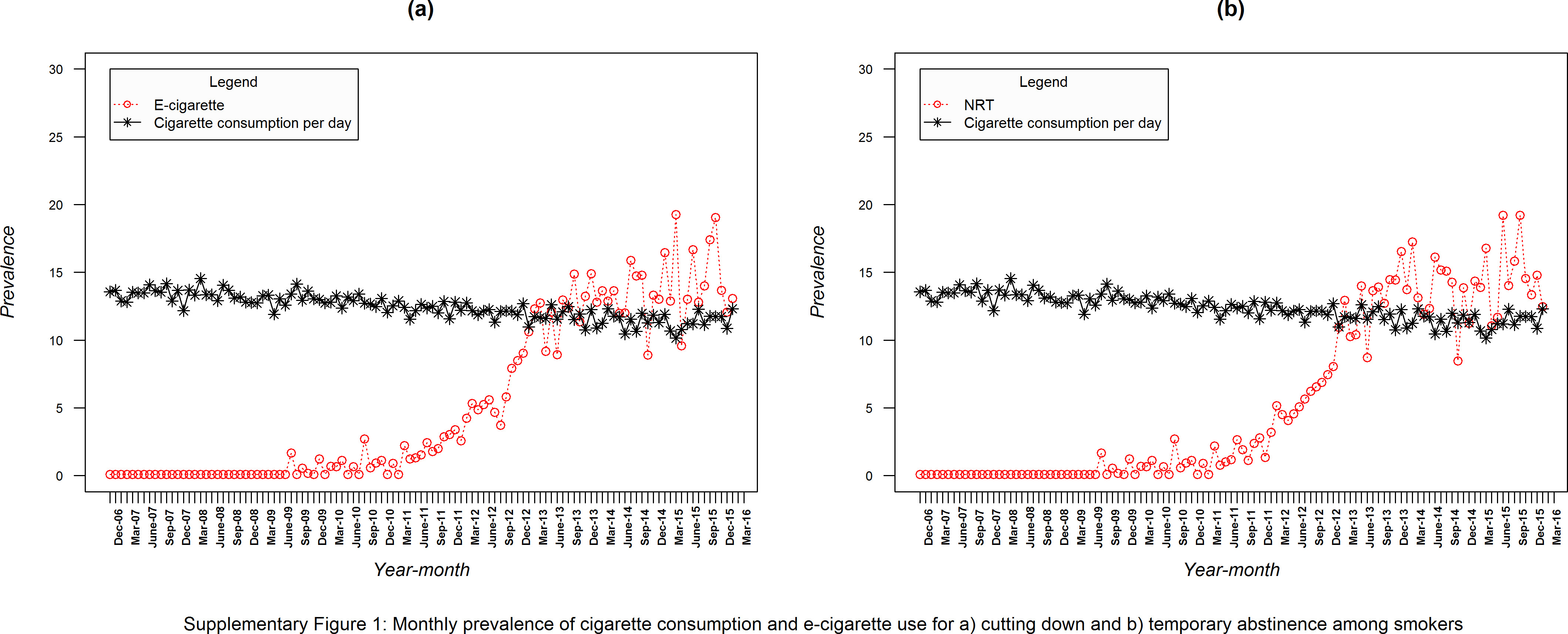

Supplement: Supplementary data [file bmjopen-2017-016046supp001.jpg]

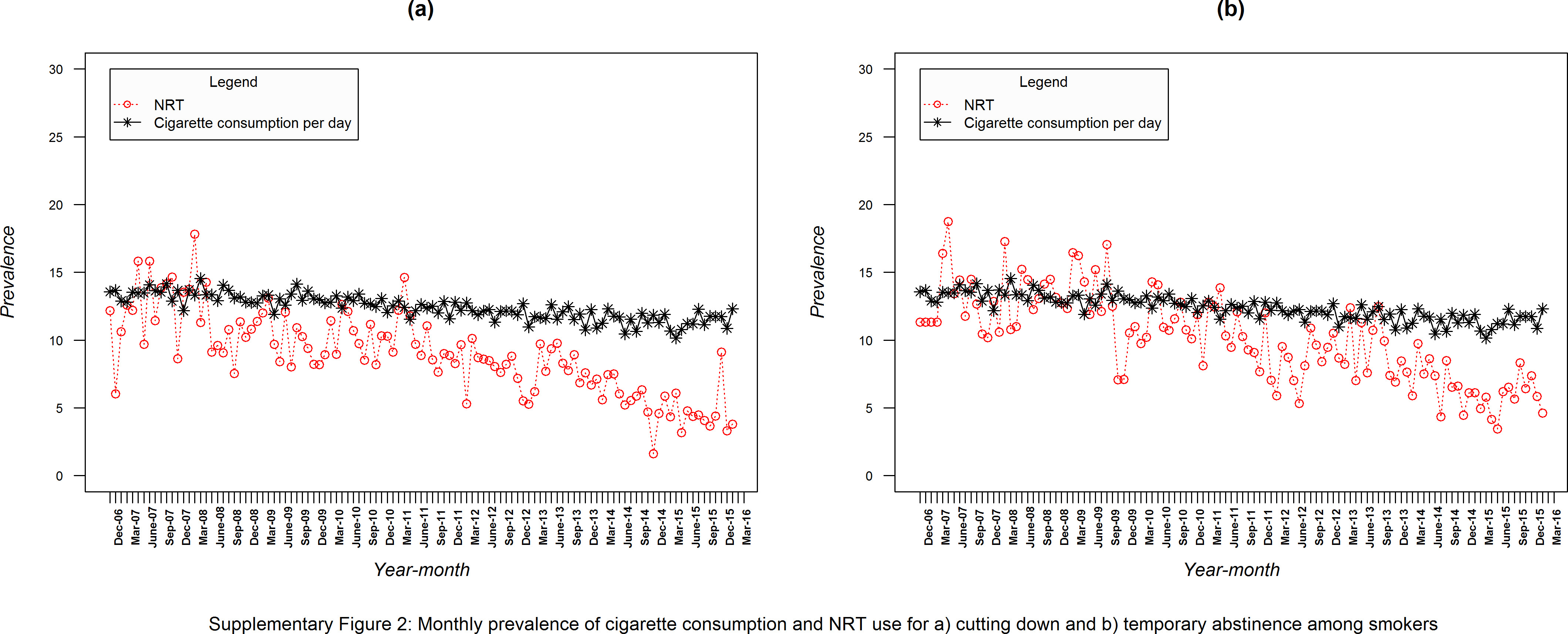

Supplement: Supplementary data [file bmjopen-2017-016046supp002.jpg]
