# Supplementary material for: Is prevalence of e-cigarette and nicotine replacement therapy use among smokers associated with average cigarette consumption in England? A time-series analysis
Source: BMJ Open. 2018 Jun 19;8(6):e016046. doi: 10.1136/bmjopen-2017-016046 (PMC6020958; doi:10.1136/bmjopen-2017-016046)
Supplement: Author's manuscript [file bmjopen-2017-016046.draft_revisions.pdf]

BMJ Open

BMJ Open is committed to open peer review. As part of this commitment we make the peer review history of every article we publish publicly available.

When an article is published we post the peer reviewers' comments and the authors' responses online. We also post the versions of the paper that were used during peer review. These are the versions that the peer review comments apply to.

The versions of the paper that follow are the versions that were submitted during the peer review process. They are not the versions of record or the final published versions. They should not be cited or distributed as the published version of this manuscript.

BMJ Open is an open access journal and the full, final, typeset and author-corrected version of record of the manuscript is available on our site with no access controls, subscription charges or pay-per-view fees (http://bmjopen.bmj.com).

If you have any questions on BMJ Open's open peer review process please email info.bmjopen@bmj.com

Association between e-cigarette and nicotine replacement therapy use among smokers with cigarette consumption in England: a time-series analysis

Journal:	*BMJ Open*
Manuscript ID	bmjopen-2017-016046
Article Type:	Research
Date Submitted by the Author:	23-Jan-2017

Complete List of Authors:	Beard, Emma; UCL,<br>Brown, Jamie; University College London, Psychology & Language Sciences<br>Michie, Susan; University College London, Centre for Outcomes Research and Effectivenes<br>West, Robert; University College London, Epidemiology and Public Health

Keywords:	Time series, ARIMAX, SMOKING, E-CIGARETTE, NRT

SCHOLARONE™
Manuscripts

Association between e-cigarette and nicotine replacement therapy use among smokers with cigarette consumption in England: a time-series analysis

Emma Beard[1,2], Jamie Brown[1,2], Susan Michie[2] & Robert West[1]

1 Cancer Research UK Health Behaviour Research Centre, University College London, WC1E 6BT, UK

2 Research Department of Educational, Clinical and Health Psychology, University College London, WC1E 7HB

For peer review only

Word count: 2843

Journal: BMJ Open

For peer review only - http://bmjopen.bmj.com/site/about/guidelines.xhtml

Correspondence to: Emma Beard, Cancer Research UK Health Behaviour Research Centre, University College London, WC1E 6BP. Email: e.beard@ucl.ac.uk. Tel: 02031083179

ABSTRACT

Objectives: Many smokers use e-cigarettes and licensed nicotine replacement therapy (NRT), often in an attempt to reduce their cigarette consumption. We estimated how far changes in prevalence of e-cigarette and NRT use while smoking was accompanied by changes in cigarette consumption at the population level.

Design: Repeated cross-sectional surveys of adults aged 16+ in England

Methods: We used ARIMAX modelling of monthly data between 2006 and 2016 from the Smoking Toolkit Study. Prevalence of e-cigarette use and NRT use in current smokers, and specifically for smoking reduction and temporary abstinence, were input variables. Mean daily cigarette consumption was the dependent variable. Analyses involved adjustment for mass media expenditure and tobacco control policies.

Results: No statistically significant associations were found between changes in use of e-cigarettes (β -0.012, 95%CI -0.026 to 0.002) or NRT (β 0.015 95%CI -0.026 to 0.055) while smoking and daily cigarette consumption. Neither did we find clear evidence for an association between e-cigarette use (β -0.010 95%CI -0.025 to 0.005 and β 0.011 95% -0.027 to 0.004) or NRT use (β 0.006 95% -0.030 to 0.043 and β 0.022 95% -0.020 to 0.063) specifically for smoking reduction and temporary abstinence respectively, and changes in daily cigarette consumption.

Conclusion: If use of e-cigarettes and licensed nicotine replacement therapy while smoking acts to reduce cigarette consumption, the effect is probably very small at a population level.

Strengths and limitations of this study

- This is the first time series study to assess the population level impact of the use of NRT and e-cigarettes for harm reduction on cigarette consumption
- This study uses a large representative sample of the population in England and considers both smoking reduction and temporary abstinence
- A wide range of confounders are adjusted for including population level interventions
- In countries with weaker tobacco control, or stricter regulation of using products for harm reduction, different effects may be observed.
- Data are observational and so strong conclusions regarding cause and effect cannot be made

For peer review only

For peer review only - http://bmjopen.bmj.com/site/about/guidelines.xhtml

INTRODUCTION

Randomised controlled trials have shown that use of non-tobacco nicotine containing products are efficacious for harm reduction attempts. Harm reduction is defined as any attempt to reduce the harm from smoking without an intention to quit completely, such as, the use of Nicotine Replacement Therapy (NRT) for smoking reduction or during periods of temporary abstinence [1]. Outside of the clinical setting where little behavioural support is provided, the use of NRT during attempts to cut down appears to increase smoker's propensity to quit, but does not result in significantly large reductions in cigarette consumption [2-4]. Explanations for this include the lack of behavioural support and possible poor compliance with the medical regimen [5 6].

In recent years, there has been an increase in the overall use of nicotine containing products for harm reduction, with a growth in e-cigarettes more than offsetting a decline in the use of licensed products [7-9]. Previous studies suggest that e-cigarettes reduce cravings more effectively than NRT [7 10 11], have better adherence rates [7 12], and deliver clinically significant levels of nicotine into the blood, at least for some smokers [10 11 13]. Thus, e-cigarettes may be a more effective aid for smoking reduction than licensed nicotine products [14 15]. However, it also remains possible that e-cigarettes will not result in clinically significant reductions in cigarette intake at a population level.

The aim of this study, using data from the Smoking Toolkit Study (STS), was to assess the association between changes in prevalence of e-cigarettes and NRT with changes in mean cigarette consumption per day using a time-series approach. Time-series analysis allows us to take into account underlying trends, the effect of other tobacco control interventions, autocorrelation (whereby data collected at points closer in time tends to be more similar), and to consider possible lag effects of the independent variable on the dependent variable [16]. Where associations are found, they cannot unequivocally establish a causal association but can be indicative, as has been the case with estimating the effect of price of cigarettes on population consumption [17], mass media expenditure on use of specialist stop smoking services [18], and introduction of varenicline to the market on prevalence of use of smoking cessation medication [19]. Where associations are not found, or they go in a direction opposite to that expected, this can also be informative.

Specifically, this paper assesses the association between mean cigarette consumption per day with:

1. E-cigarette use among smokers
2. NRT use among smokers

In both instances, three models will be assessed separately assessing current use for any purpose, current use specifically for smoking reduction, and current use specifically for

For peer review only - http://bmjopen.bmj.com/site/about/guidelines.xhtml

For peer review only

temporary abstinence. Sensitivity analyses will examine the effect of restricting analyses to focus on daily e-cigarette and NRT use, given previous associations between extent of non-tobacco nicotine containing product use and the effectiveness of harm reduction attempts [6].

METHODS

Data sources

Data on explanatory variables

The STS is a monthly survey of a representative sample of the population in England aged 16+ [20]. This has been collecting data on smoking patterns among smokers and recent ex-smokers since November 2006. Questions on the use of e-cigarettes among all smokers were introduced in May 2011 and as aids to a quit attempt among smokers attempting to stop in July 2009. The STS involves monthly household surveys using a random location sampling design, with initial random selection of grouped output areas (containing 300 households), stratified by ACORN (sociodemographic) characteristics (http://www.caci.co.uk/acron/acornmap.asp) and region. Interviewers then choose which houses within these areas are most likely to fulfil quotas based on the probability of being at home tailored to region and conduct face-to-face computer assisted interviews with one member per household. Participants from the STS appear to be representative of the population in England, having similar socio-demographic composition as other large national surveys, such as the Health Survey for England [20].

Participants who reported that they smoked cigarettes (including hand-rolled) every day or that they smoked cigarettes (including hand-rolled) but not every day, were asked the following questions:

1. Which, if any, of the following are you currently using to help you cut down the amount you smoke? Answers: nicotine gum, nicotine replacement lozenges\tablets, nicotine replacement inhaler, nicotine replacement nasal spray, nicotine patch, electronic cigarette, nicotine mouth spray, other.
2. Do you regularly use any of the following in situations when you are not allowed to smoke? Answer: nicotine gum, nicotine replacement lozenges\tablets, nicotine replacement inhaler, nicotine replacement nasal spray, nicotine patch, electronic cigarette, nicotine mouth spray, other.
3. Can I check, are you using any of the following either to help you stop smoking, to help you cut down or for any other reason at all? Answer: nicotine gum, nicotine replacement lozenges\tablets, nicotine replacement inhaler, nicotine replacement nasal spray, nicotine patch, electronic cigarette, nicotine mouth spray, other.

Smokers answering *electronic* cigarettes to any of the three questions were categorised as current e-cigarette users, while smokers answering an *NRT* product were classed as current NRT users. Smokers answering *electronic* cigarettes and *NRT* to the first question were categorised as using e-cigarettes and *NRT* for smoking reduction respectively, while those answering *electronic* cigarettes and *NRT* to the second question were categorised as using *e-cigarettes* and *NRT* for temporary abstinence respectively. Data were not recorded on NRT use for temporary abstinence between Nov-2006 and Jan-2007 and was imputed using prevalence data from Feb-2007.

Data were only available on the prevalence of use of electronic cigarettes among smokers from April 2011 – although use specifically during a recent quit attempt were available from July 2009. Thus, prevalence of electronic cigarette use among smokers between July 2009

For peer review only - http://bmjopen.bmj.com/site/about/guidelines.xhtml

and April 2011 was estimated from data on use during a quit attempt; use of electronic cigarettes among smokers between November 2006 and June 2009 was assumed to be 0.1% of smokers based on other surveys, which found their use to be very rare before 2009 [21 22].

Daily NRT and e-cigarette users were classified as those who reported that they used the product(s) at least once per day in response to the question: How many times per day on average do you use your nicotine replacement product or products? This question was introduced in July-2010. Prior to this time, prevalence of daily NRT use was assumed to be 60% of all users [6], while e-cigarette prevalence was computed as above using prevalence during a quit attempt or 0.1%.

Data on outcome variables

Smokers taking part in the STS were also asked how many cigarettes they smoke on average per day.

Data on other co-variables

In England, tobacco mass media campaigns have been run as part of a national tobacco control programme. Spending was almost completely suspended in 2010 and then re-introduced in 2011 at a much lower level. Previous studies have shown that such cuts were associated with a decreased use of smoking cessation support [18 23]. Thus, advertising expenditure will be adjusted for using data obtained from Public Health England. Data on mass media expenditure was available monthly from May 2008, and yearly prior to this period, and so a monthly average was assumed. For a number of months spending was effectively zero and was imputed as 0.1 to allow the analysis to run.

A number of tobacco control policies were adjusted for. These included the move in commissioning of stop smoking services to local authorities in April 2013 [24], introduction of a smoking ban in July 2007 [25], licensing of NRT for harm reduction in December 2009 [26], the publication of NICE guidance on harm reduction in June 2013 [27], and change in the minimum age of sale of cigarettes October 2007 [28]. Price of cigarettes is correlated 0.99 with time and will thereby be taken into account by use of differencing to make the series stationary.

ANALYSIS

The analysis plan was registered on the Open Science Framework prior to data analysis (https://osf.io/6swk3/). All data were analysed in R version 3.2.4 [29] using Autoregressive Integrated Moving Average with Exogeneous Input (ARIMAX) modelling [16 30 31]. Data were weighted prior to the analyse to match the population in England (for more details see [20]).

Two waves of data were collected in March 2007 and March 2013, these waves were averaged. No data were collected in December 2008. Mean cigarette consumption, NRT use and e-cigarette use during this period was calculated as an average of the month before and the month after. For a few months (May-2012, Jul-2012, Sep-2012, Nov-2012, Jan-2013, Mar-2013), data on electronic cigarettes and NRT use among smokers were not recorded. For these months, the average of the previous and next month was imputed.

The Granger causality test suggested that there was some evidence for the violation of the assumption of weak exogeneity (i.e. Y can depend on the lagged values of X but the reverse must not be true) between the input and the output series. However, caution has been advised when using this and similar tests on data across a long time-series [32 33] and there

For peer review only - http://bmjopen.bmj.com/site/about/guidelines.xhtml

was no theoretical reason we could identify for a bi-directional relationship between e-cigarette use and cigarette consumption. It was assumed that the association was spurious and likely removed following adjustment for other covariates.

Both unadjusted and fully adjusted models are reported which regressed onto mean cigarette consumption per day 1) use of e- cigarettes among current smokers; 2) use of e-cigarettes for smoking reduction; 3) use of e- cigarettes for temporary abstinence; 4) use of NRT for harm reduction; 5) use of NRT for temporary abstinence and 6) use of NRT for smoking reduction. Sensitivity analyses were conducted which constrained the analysis to only those reporting daily e-cigarette and NRT use. We followed a standard ARIMAX modelling approach [16 34]. The series were first log-transformed to stabilise the variance, and if required, first differenced and seasonally differenced. The autocorrelation and partial autocorrelation functions were then examined in order to determine the seasonal and non-seasonal moving average (MA) and autoregressive terms (AR). To identify the most appropriate transfer function for the continuous explanatory variables the sample cross-correlation function was checked for each ARIMAX model. Coefficients can be interpreted as estimates of the percentage change in cigarette consumption for every a) percentage increase in use of e-cigarettes and NRT, b) percentage increase in mass media expenditure, and c) implementation of tobacco control policies.

Bayes factors (BF) were derived for non-significant findings using an online calculator [35] to disentangle whether there is evidence for the null hypothesis of no effect (Bayes Factor <1/3rd) or the data are insensitive (Bayes Factor between 1/3rd and 3). A half-normal distribution was assumed with a percentage change in the outcomes of interest for every percentage increase in the input series of 0.009% based on the effect detectable with 80% power (see sample size). Sensitivity analyses were conducted using a much larger percentage change of 0.1. This was based on a meta-analysis assessing the efficacy of non-tobacco nicotine replacement products for harm reduction, which reported that 21.8% of the experimental group had reduced consumption by more than 50% at final follow-up compared with 16.5% receiving placebo [1]. We therefore assumed that a 5% change in prevalence of NRT and e-cigarettes would be associated with a 0.5% change in overall cigarette consumption.

STROBE guidelines for the reporting of observational studies were followed throughout [36].

Sample size

Simulation-based power analyses suggested that this study would have 80% power to detect a change in the output series of 0.009% for every 1% change in the input series, assuming 113 monthly data collection points, MA(1) autocorrelation [37], a baseline proportion for the input series of 0.005 [9], a baseline mean (SD) for the output series of 12.3 [38], and a total change over time for the input series of 30% [38].

RESULTS

Sample characteristics

Data were collected on 199,483 adults aged 16+ taking part in the STS who reported their smoking status between November 2006 and March 2016. Of these, 43,608 (20.8%: 95%CI 20.6 to 21.0) were current smokers. Fifty-two percent (95%CI 52.0 to 53.0) of smokers were male and 60.4% (95%CI 60.0 to 60.1%) were in routine or manual positions or were unemployed. The average age of smokers in this study was 42.1 years (95%CI 42.0 to 42.1)

Main analysis

Figure 1 shows that current use of e-cigarettes among smokers for harm reduction increased from negligible use in the last quarter of 2006 to 17.1% at the end of the study (mean 7.8%,

For peer review only - http://bmjopen.bmj.com/site/about/guidelines.xhtml

SD 8.82). Figure 2 shows that there was also a decline in the use of NRT for harm reduction from 12.2% to 6.0% (mean 14.4%, SD 4.36). Cigarette consumption also declined over the study period from 13.6 to 12.3 (mean 12.4, SD 0.92). Supplementary Figures 1 and 2 show the changes in e-cigarette and NRT use for smoking reduction and temporary abstinence, respectively.

Tables 2, 3 and 4 show the results of the ARIMAX models assessing the association between cigarette consumption per day with 1) e- cigarette use among current smokers and NRT use for harm reduction; 2) e- cigarette and NRT use for smoking reduction and 3) e-cigarette and NRT use for temporary abstinence. The findings were inconclusive as to whether or not an association was present between use of e-cigarettes and NRT for any purpose and cigarette consumption.

Bayes Factors were between 1/3rd and 3 when assuming a 0.009% change in cigarette consumption for every percentage change in the input series, suggesting the data are insensitive to detect very small reductions in cigarette consumption. Most Bayes Factors were <1/3rd, when assuming a 0.1% change in cigarette consumption for every percentage change in the input series, suggesting evidence for the null hypothesis that NRT use and e-cigarette use among smokers has not resulted in large reductions in cigarette intake.

For peer review only

For peer review only - http://bmjopen.bmj.com/site/about/guidelines.xhtml

Sensitivity analysis

Current daily use of e-cigarettes among smokers for harm reduction increased from negligible use in the last quarter of 2006 to 11.1% at the end of the study (mean 4.5%, SD 4.91). There was also an increase in e-cigarette use specifically for temporary abstinence (from 0.1% to 8.4%; mean 3.5% SD 3.81) and smoking reduction (from 0.1% to 8.3%; mean 3.3% SD 3.64).

In contrast, there was a decline in the use of NRT for harm reduction from 7.3% to 2.9% (mean 6.5%, SD 2.35) and a decline in NRT use specifically for temporary abstinence (from 7.3% to 1.8%; mean 4.7% SD 2.29) and smoking reduction (from 6.8% to 2.6%; 5.8% SD 2.46).

Tables 2, 3 and 4 also show the results of the sensitivity analyses restricted to those smokers using NRT or e-cigarettes daily. The findings were inconclusive as to whether or not an association was present between the daily use of e- cigarettes and NRT for any purpose and cigarette consumption. Bayes Factors suggested the data are insensitive to detect very small reductions in cigarette consumption, but there is evidence for the null hypothesis that NRT use and e-cigarette use among smokers has not resulted in large reductions in cigarette intake.

DISCUSSION

To our knowledge, this is the first empirical study to estimate the population association between the use of e-cigarettes and NRT among current smokers on cigarette consumption per day, using a time-series approach. There was evidence that there was no substantial association between the rise in use of e-cigarettes and decline in NRT use and changes in cigarette consumption per day.

A strength of the study is the use of a large representative sample of the population in England, stratification of results by daily use, and the consideration of both temporary abstinence and smoking reduction. Previous studies have shown that reductions in cigarette intake are dependent on the extent of NRT use and differ as a function of the specific harm reduction behaviour i.e. an attempt to cut down or restraining from smoking during periods of brief abstinence [2 6].

The study had a number of limitations. First, caution should be taken when interpreting estimates of the covariates, i.e. impact of some of the tobacco control policies, as interrupted explanatory variables with short time-periods prior to their introduction in ARIMA type models often give inaccurate estimates of the standard errors [28]. Thus, although the increase in age-of-sale has been previously associated with a decline in smoking prevalence [24], the short lead in period may have masked any true association [27]. Secondly, the STS required participants to recall their average daily cigarette intake which is likely to have been somewhat inaccurate. Thirdly, the findings may not generalise to other countries. England has a strong tobacco control climate and relatively liberal attitude towards harm reduction and e-cigarette use. In countries with weaker tobacco control, or stricter regulation of using products for harm reduction, different effects may be observed. Finally, although we are unaware of any other major population level interventions or other events during the study period, we cannot rule out residual confounding.

The findings are in line with previous studies which show that reductions in cigarette consumption observed in clinical trials of NRT for harm reduction do not appear to generalise beyond the closely controlled trial setting [1 2]. It was hypothesised that e-cigarettes may be associated with population mean cigarette intake given that they reduce cravings more effectively than NRT [7 10 11], have better adherence rates [7 12], and deliver clinically significant levels of nicotine into the blood [10 11 13]. Of course, it remains

For peer review only - http://bmjopen.bmj.com/site/about/guidelines.xhtml

plausible that e-cigarettes may still be associated with a very small effect on mean population cigarette consumption [15], and that a reduction in harm from smoking at a population level could be seen through their promotion of quit attempts [37] or by reducing smoke intake from each cigarette [5].

In conclusion, the increased prevalence of e-cigarettes use among smokers in England has not been associated with a detectable change in cigarette consumption per day. The decline in the use of NRT has also not been associated with a change in mean cigarette intake. If use of e-cigarettes and licensed nicotine replacement therapy while smoking acts to reduce cigarette consumption, the effect is probably small.

Declaration of interests

RW undertakes consultancy and research for and receives travel funds and hospitality from manufacturers of smoking cessation medications but does not, and will not take funds from e-cigarettes manufacturers or the tobacco industry. RW and SM are honorary co-directors of the National Centre for Smoking Cessation and Training. RW is a Trustee of the stop-smoking charity, QUIT. RW salary is funded by Cancer Research UK. SM salary is funded by Cancer Research UK and by the National Institute for Health Research (NIHR)'s School for Public Health Research (SPHR). EB and JB have received unrestricted research funding from Pfizer. EB and JB are funded by CRUK. EB is also funded by NIHR's SPHR and JB by the Society for the Study of Addiction. RW has received travel funds and hospitality from, and undertaken research and consultancy for pharmaceutical companies that manufacture or research products aimed at helping smokers to stop. These products include nicotine replacement therapies, Champix (varenicline) and Zyban (bupropion). This has led to payments to him personally and to his institution.

Author contributions

EB, JB, SM and RW designed the study. EB wrote the first draft and conducted the analyses. All authors commented on this draft and contributed to the final version.

Data sharing

For access to the data please contact the lead author, Dr Emma Beard e.beard@ucl.ac.uk

Sources of funding

The Smoking Toolkit Study is currently primarily funded by Cancer Research UK (C1417/A14135; C36048/A11654; C44576/A19501) and has previously also been funded by Pfizer, GSK, and the Department of Health. JB's post is funded by a fellowship from the Society for the Study of Addiction and CRUK also provide support (C1417/A14135); RW is funded by Cancer Research UK (C1417/A14135); EB is funded by a fellowship from the NIHR SPHR (SPHR-SWP-ALC-WP5) and CRUK also provide support (C1417/A14135). SW is funded by Cancer Research UK (C1417/A14135) and NIHR SPHR (SPHR-SWP-ALC-WP5) also provide support. SPHR is a partnership between the Universities of Sheffield; Bristol; Cambridge; Exeter; UCL; The London School for Hygiene and Tropical Medicine; the LiLaC collaboration between the Universities of Liverpool and Lancaster and Fuse; The Centre for Translational Research in Public Health, a collaboration between Newcastle, Durham, Northumbria, Sunderland and Teesside Universities. The views expressed are those of the authors(s) and not necessarily those of the NHS, NIHR, or Department of Health. No funders had any involvement in the design of the study, the analysis or interpretation of the data, the writing of the report, or the decision to submit the paper for publication

For peer review only - http://bmjopen.bmj.com/site/about/guidelines.xhtml

Table 1: Estimated percentage point changes in mean cigarette consumption per day as a function of e- cigarette use and NRT use among smokers from November 2006 until March 2016, based on Autoregressive Integrated Moving Average with Exogeneous Input (ARIMAX) models

	**All users of nicotine replacement**			**Only daily users of nicotine replacement**		
	Percentage change per 1% change in the exposure (95%CI) p	Percentage change per 1% change in the exposure (95%CI) p	Percentage change per 1% change in the exposure (95%CI) P	Percentage change per 1% change in the exposure (95%CI) p	Percentage change per 1% change in the exposure (95%CI) p	Percentage change per 1% change in the exposure (95%CI) p
Any current use of e- cigarettes (Immediate impact)	-0.011 (-0.025 to 0.002) 0.097		-0.012 (-0.026 to 0.002) 0.091	-0.010 (-0.024 to 0.004) 0.149		-0.011 (-0.026 to 0.003) 0.130
NRT use for harm reduction (Immediate impact)		0.012 (-0.028 to 0.053) 0.546	0.015 (-0.026 to 0.055) 0.475		0.003 (-0.019 to 0.025) 0.794	0.005 (-0.017 to 0.027) 0.672
Mass media expenditure (Immediate impact)			<0.001 (-0.001 to 0.001) 0.984			<0.001(-0.001 to 0.001) 0.880
	Total percentage change due to the exposure (95%CI) p	Total percentage change due to the exposure (95%CI) p	Total percentage change due to the exposure (95%CI) P	Total percentage change due to the exposure (95%CI) p	Total percentage change due to the exposure (95%CI) P	Total percentage change due to the exposure (95%CI) P
Smoking ban (Pulse effect)			0.015 (-0.070 to 0.101) 0.724			0.013 (-0.072 to 0.099) 0.756
Increase in age-of-sale (Pulse effect)			-0.041 (-0.126 to 0.044) 0.342			-0.043 (-0.128 to 0.042) 0.324
Move to local authority control (Pulse effect)			-0.019 (-0.105 to 0.067) 0.662			-0.027 (-0.112 to 0.058) 0.533
Licensing for NRT for harm reduction (Pulse effect)			0.021 (-0.067 to 0.110) 0.639			0.020 (-0.069 to 0.109) 0.661
NICE guidance on harm reduction (Pulse effect)			-0.024 (-0.109 to 0.061) 0.578			-0.028 (-0.114 to 0.057) 0.512
Best fitting model	ARIMA(0,1,1)(0,0,0)[12]	ARIMA(0,1,1)(0,0,0)[12]	ARIMA(0,1,1)(0,0,0)[12]	ARIMA(0,1,1)(0,0,0)[12]	ARIMA(0,1,1)(0,0,0)[12]	ARIMA(0,1,1)(0,0,0)[12]
Non-seasonal AR *p*	NA	NA	NA	NA	NA	NA
Non-seasonal MA *p*	<0.001	<0.001	<0.001	<0.001	<0.001	<0.001
Seasonal AR *p*	NA	NA	NA	NA	NA	NA
Seasonal MA *p*	NA	NA	NA	NA	NA	NA
R-squared	0.65	0.65	0.66	0.65	0.64	0.66
**Bayes Factor e-cigarette [0.009(0.1)]**	2.44 (0.46)		2.68 (0.55)	1.95 (0.35)		2.12 (0.41)
**Bayes Factor NRT [0.009(0.1)]**		0.77 (0.14)	0.74 (0.13)		0.69 (0.09)	0.63 (0.08)

Note: 95%CI=95% confidence interval; MA=moving average; AR=autoregressive; NA=not applicable; An AR(1) means that the value of a series at one point in time is the sum of a fraction of the value of the series at the immediately preceding point in time and an error component; An MA(1) means that the value of a series at one point in time is a function of a fraction of the error component of the series at the immediately preceding point in time and an error component at the current point in time.

For peer review only - http://bmjopen.bmj.com/site/about/guidelines.xhtml

Table 2: Estimated percentage point changes in mean cigarette consumption per day as a function of e- cigarette use and NRT use among smokers for cutting down from November 2006 until March 2016, based on Autoregressive Integrated Moving Average with Exogeneous Input (ARIMAX) models

	**All users of nicotine replacement**			**Only daily users of nicotine replacement**		
	Percentage change per 1% change in the exposure (95%CI) P	Percentage change per 1% change in the exposure (95%CI) p	Percentage change per 1% change in the exposure (95%CI) P	Percentage change per 1% change in the exposure (95%CI) P	Percentage change per 1% change in the exposure (95%CI) P	Percentage change per 1% change in the exposure (95%CI) P
Use of e- cigarettes for cutting down (Immediate impact)	-0.010 (-0.024 to 0.005) 0.191		-0.010 (-0.025 to 0.005) 0.191	-0.008 (-0.023 to 0.006) 0.256		-0.009 (-0.024 to 0.006) 0.229
NRT use for cutting down (Immediate impact)		0.002 (-0.033 to 0.037) 0.917	0.006 (-0.030 to 0.043) 0.732		-0.002 (-0.016 to 0.013) 0.825	-0.002 (-0.017 to 0.013) 0.786
Mass media expenditure (Immediate impact)			<0.001 (-0.001 to 0.001) 0.885			<0.001 (-0.001 to 0.001) 0.860
	Total percentage change due to the exposure (95%CI) P	Total percentage change due to the exposure (95%CI) p	Total percentage change due to the exposure (95%CI) P	Total percentage change due to the exposure (95%CI) P	Total percentage change due to the exposure (95%CI) P	Total percentage change due to the exposure (95%CI) P
Smoking ban (Pulse effect)			0.014 (-0.072 to 0.099) 0.755			0.012 (-0.073 to 0.097) 0.782
Increase in age-of-sale (Pulse effect)			-0.043 (-0.128 to 0.042) 0.323			-0.042 (-0.127 to 0.043) 0.329
Move to local authority control (Pulse effect)			-0.025 (-0.110 to 0.061) 0.571			-0.029 (-0.115 to 0.056) 0.499
Licensing for NRT for harm reduction (Pulse effect)			0.018 (-0.072 to 0.108) 0.694			0.015 (-0.074 to 0.103) 0.747
NICE guidance on harm reduction (Pulse effect)			-0.028 (0.058 to <0.001) 0.529			-0.027 (-0.112 to 0.059) 0.541
Best fitting model	ARIMA(0,1,1)(0,0,0)^{12}	ARIMA(0,1,1)(0,0,0)^{12}	ARIMA(0,1,1)(0,0,0)^{12}	ARIMA(0,1,1)(0,0,0)^{12}	ARIMA(0,1,1)(0,0,0)^{12}	ARIMA(0,1,1)(0,0,0)^{12}
Non-seasonal AR *p*	NA	NA	NA	NA	NA	NA
Non-seasonal MA *p*	<0.001	<0.001	<0.001	<0.001	<0.001	<0.001
Seasonal AR *p*	NA	NA	NA	NA	NA	NA
Seasonal MA *p*	NA	NA	NA	NA	NA	NA
R-squared	0.64	0.64	0.65	0.64	0.64	0.65
**Bayes Factor e-cigarette [0.009(0.1)]**	1.87 (0.34)		1.79 (0.32)	1.46 (0.23)		1.61 (0.27)
**Bayes Factor NRT [0.009(0.1)]**		0.86 (0.16)	0.81 (0.15)		0.76 (0.10)	0.76 (0.10)

Note: 95%CI=95% confidence interval; MA=moving average; AR=autoregressive; NA=not applicable; An AR(1) means that the value of a series at one point in time is the sum of a fraction of the value of the series at the immediately preceding point in time and an error component; An MA(1) means that the value of a series at one point in time is a function of a fraction of the error component of the series at the immediately preceding point in time and an error component at the current point in time.

For peer review only

For peer review only - http://bmjopen.bmj.com/site/about/guidelines.xhtml

Table 3: Estimated percentage point changes in mean cigarette consumption per day as a function of e- cigarette use and NRT use among smokers for temporary abstinence from November 2006 until March 2016, based on Autoregressive Integrated Moving Average with Exogeneous Input (ARIMAX) models

	**All users of nicotine replacement**			**Only daily users of nicotine replacement**		
	Percentage change per 1% change in the exposure (95%CI) P	Percentage change per 1% change in the exposure (95%CI) p	Percentage change per 1% change in the exposure (95%CI) P	Percentage change per 1% change in the exposure (95%CI) p	Percentage change per 1% change in the exposure (95%CI) p	Percentage change per 1% change in the exposure (95%CI) P
Use of e- cigarettes for temporary abstinence (Immediate impact)	-0.010 (-0.024 to 0.005) 0.150		-0.011 (-0.027 to 0.004) 0.146	-0.010 (-0.024 to 0.004) 0.159		-0.011 (-0.026 to 0.003) 0.135
NRT use for temporary abstinence (Immediate impact)		0.023 (-0.016 to 0.062) 0.241	0.022 (-0.020 to 0.063) 0.303		0.006 (-0.015 to 0.028) 0.563	0.006 (-0.016 to 0.028) 0.585
Mass media expenditure (Immediate impact)			<0.001 (-0.001 to 0.001) 0.873			<0.001 (-0.001 to 0.001) 0.942
	Total percentage change due to the exposure (95%CI) P	Total percentage change due to the exposure (95%CI) P	Total percentage change due to the exposure (95%CI) P	Total percentage change due to the exposure (95%CI) p	Total percentage change due to the exposure (95%CI) P	Total percentage change due to the exposure (95%CI) P
Smoking ban (Pulse effect)			0.017 (-0.069 to 0.103) 0.696			0.014 (-0.071 to 0.099) 0.750
Increase in age-of-sale (Pulse effect)			-0.036 (-0.122 to 0.050) 0.415			-0.040 (-0.125 to 0.044) 0.350
Move to local authority control (Pulse effect)			-0.016 (-0.102 to 0.071) 0.721			-0.026 (-0.111 to 0.060) 0.556
Licensing for NRT for harm reduction (Pulse effect)			0.023 (-0.067 to 0.114) 0.615			0.019 (-0.070 to 0.108) 0.670
NICE guidance on harm reduction (Pulse effect)			-0.021 (-0.106 to 0.065) 0.638			-0.030 (-0.116 to 0.055) 0.483
Best fitting model	ARIMA(0,1,1)(0,0,0)^{12}	ARIMA(0,1,1)(0,0,0)^{12}	ARIMA(0,1,1)(0,0,0)^{12}	ARIMA(0,1,1)(0,0,0)^{12}	ARIMA(0,1,1)(0,0,0)^{12}	ARIMA(0,1,1)(0,0,0)^{12}
Non-seasonal AR *p*	NA	NA	NA	NA	NA	NA
Non-seasonal MA *p*	<0.001	<0.001	<0.001	<0.001	<0.001	<0.001
Seasonal AR *p*	NA	NA	NA	NA	NA	NA
Seasonal MA *p*	NA	NA	NA	NA	NA	NA
R-squared	0.65	0.65	0.65	0.65	0.64	0.65
**Bayes Factor e-cigarette [0.009(0.1)]**	1.01 (0.59)		1.94 (0.38)	1.97 (0.35)		2.15 (0.41)
**Bayes Factor NRT [0.009(0.1)]**		0.15 (0.02)	0.69 (0.11)		1.05 (0.18)	0.61 (0.08)

Note: 95%CI=95% confidence interval; MA=moving average; AR=autoregressive; NA=not applicable; An AR(1) means that the value of a series at one point in time is the sum of a fraction of the value of the series at the immediately preceding point in time and an error component; An MA(1) means that the value of a series at one point in time is a function of a fraction of the error component of the series at the immediately preceding point in time and an error component at the current point in time.

For peer review only - http://bmjopen.bmj.com/site/about/guidelines.xhtml

References

1. Moore D, Aveyard P, Connock M, et al. Effectiveness and safety of nicotine replacement therapy assisted reduction to stop smoking: systematic review and meta-analysis. Bmj 2009;**338**:b1024
2. Beard E, McNeill A, Aveyard P, et al. Use of nicotine replacement therapy for smoking reduction and during enforced temporary abstinence: a national survey of English smokers. Addiction 2011;**106** doi: 10.1111/j.1360-0443.2010.03215.x[published Online First: Epub Date]|.
3. Beard E, Michie S, Fidler J, et al. Use of nicotine replacement therapy in situations involving temporary abstinence from smoking: a national survey of English smokers. Addictive behaviors 2013;**38**(3):1876-79
4. Beard E, Aveyard P, Michie S, et al. Does use of nicotine replacement therapy while continuing to smoke undermine cessation?: a systematic review. Journal of Smoking Cessation 2013;**8**(01):45-56
5. Beard E, Vangeli E, Michie S, et al. The use of nicotine replacement therapy for smoking reduction and temporary abstinence: an interview study. Nicotine & Tobacco Research 2012;**14**(7):849-56
6. Beard E, Bruguera C, McNeill A, et al. Association of amount and duration of NRT use in smokers with cigarette consumption and motivation to stop smoking: A national survey of smokers in England. Addictive behaviors 2015;**40**:33-38
7. Etter JF, Bullen C. Electronic cigarette: users profile, utilization, satisfaction and perceived efficacy. Addiction 2011;**106**(11):2017-28
8. Etter J-F. Electronic cigarettes: a survey of users. BMC public health 2010;**10**(1):1
9. Beard E, Brown J, McNeill A, et al. Has growth in electronic cigarette use by smokers been responsible for the decline in use of licensed nicotine products? Findings from repeated cross-sectional surveys. Thorax 2015:thoraxjnl-2015-206801
10. Vansickel AR, Cobb CO, Weaver MF, et al. A clinical laboratory model for evaluating the acute effects of electronic "cigarettes": nicotine delivery profile and cardiovascular and subjective effects. Cancer Epidemiology Biomarkers & Prevention 2010;**19**(8):1945-53
11. Bullen C, Howe C, Laugesen M, et al. Electronic cigarettes for smoking cessation: a randomised controlled trial. The Lancet 2013;**382**(9905):1629-37
12. Kralikova E, Kubatova S, Truneckova K, et al. The electronic cigarette: what proportion of smokers have tried it and how many use it regularly? Addiction 2012;**107**(8):1528-29
13. Dawkins L. Electronic cigarettes: what are they and are they effective? E-Cigarette Summit, London, UK: (oral presentation). Secondary Electronic cigarettes: what are they and are they effective? E-Cigarette Summit, London, UK: (oral presentation). 2013. http://e-cigarette-summit.com/wp-content/uploads/2013/12/Summit-Presentations.pdf.
14. Polosa R, Caponnetto P, Morjaria JB, et al. Effect of an electronic nicotine delivery device (e-Cigarette) on smoking reduction and cessation: a prospective 6-month pilot study. BMC public health 2011;**11**(1):1
15. McRobbie H, Bullen C, Hartmann-Boyce J, et al. Electronic cigarettes for smoking cessation and reduction. Cochrane Database Syst Rev 2014;**12**
16. Box GE, Jenkins GM, Reinsel GC. *Time series analysis: forecasting and control*: John Wiley & Sons, 2011.
17. Gallus S, Schiaffino A, Vecchia CL, et al. Price and cigarette consumption in Europe. Tobacco Control 2006;**15**(2):114-19 doi: 10.1136/tc.2005.012468[published Online First: Epub Date]|.
18. Langley T, Szatkowski L, Lewis S, et al. The freeze on mass media campaigns in England: a natural experiment of the impact of tobacco control campaigns on quitting behaviour. Addiction 2014;**109**(6):995-1002

For peer review only - http://bmjopen.bmj.com/site/about/guidelines.xhtml

19. Langley TE, Huang Y, McNeill A, et al. Prescribing of smoking cessation medication in England since the introduction of varenicline. Addiction 2011;**106**(7):1319-24 doi: 10.1111/j.1360-0443.2011.03426.x[published Online First: Epub Date]|.
20. Fidler JA, Shahab L, West O, et al. 'The smoking toolkit study': a national study of smoking and smoking cessation in England. BMC Public Health 2011;**11**(1):479
21. Regan AK, Promoff G, Dube SR, et al. Electronic nicotine delivery systems: adult use and awareness of the 'e-cigarette'in the USA. Tobacco control 2013;**22**(1):19-23
22. Cho JH, Shin E, Moon S-S. Electronic-cigarette smoking experience among adolescents. Journal of Adolescent Health 2011;**49**(5):542-46
23. Wakefield MA, Durkin S, Spittal MJ, et al. Impact of tobacco control policies and mass media campaigns on monthly adult smoking prevalence. American Journal of Public Health 2008;**98**(8):1443-50
24. Health and Social Care Information Centre. NHS Stop Smoking Services Collection. Secondary NHS Stop Smoking Services Collection 2015. www.hscic.gov.uk/stopsmoking.
25. Hackshaw L, McEwen A, West R, et al. Quit attempts in response to smoke-free legislation in England. Tobacco control 2010;**19**(2):160-64
26. Beard E, Bruguera C, Brown J, et al. Was the Expansion of the Marketing License for Nicotine Replacement Therapy in the United Kingdom to Include Smoking Reduction Associated With Changes in Use and Incidence of Quit Attempts? Nicotine & Tobacco Research 2013;**15**(10):1777-81 doi: 10.1093/ntr/ntt044[published Online First: Epub Date]|.
27. NICE. NICE guidelines [PH45]: Smoking: Harm reduction. Secondary NICE guidelines [PH45]: Smoking: Harm reduction 2013. https://www.nice.org.uk/guidance/ph45.
28. Fidler JA, West R. Changes in smoking prevalence in 16–17-year-old versus older adults following a rise in legal age of sale: findings from an English population study. Addiction 2010;**105**(11):1984-88
29. R Development Core Team. R: A language and environment for statistical computing. R foundation for statistical computing, Vienna, Austria. Secondary R: A language and environment for statistical computing. R foundation for statistical computing, Vienna, Austria. 2008. http://www.R-project.org.
30. Wakefield MA, Coomber K, Durkin SJ, et al. Time series analysis of the impact of tobacco control policies on smoking prevalence among Australian adults, 2001? 2011. Bulletin of the World Health Organization 2014;**92**(6):413-22
31. Cryer JD, Chan K-S. *Time series analysis - with applications in R*. London: Springer-Verlag New York, 2008.
32. Yalta AT. Analyzing energy consumption and GDP nexus using maximum entropy bootstrap: The case of Turkey. Energy Economics 2011;**33**(3):453-60
33. Granger CW. Some recent development in a concept of causality. Journal of econometrics 1988;**39**(1):199-211
34. Box GE, Tiao GC. Intervention analysis with applications to economic and environmental problems. Journal of the American Statistical association 1975;**70**(349):70-79
35. Dienes Z. Using Bayes to get the most out of non-significant results. Frontiers in psychology 2014;**5**
36. Von Elm E, Altman DG, Egger M, et al. The Strengthening the Reporting of Observational Studies in Epidemiology (STROBE) statement: guidelines for reporting observational studies. Preventive medicine 2007;**45**(4):247-51
37. Beard E, Brown J, Michie S, et al. Association between population changes in use of electronic cigarettes and changes in quit attempts, use of smoking cessation pharmacotherapy and use of stop smoking services: a time-series analysis. Under-prepartion
38. Kuipers MAG, Beard E, Hitchman SC, et al. Impact on smoking of England's 2012 partial tobacco point of sale display ban: a repeated cross-sectional national study. Tobacco Control 2016 doi: 10.1136/tobaccocontrol-2015-052724[published Online First: Epub Date]|.

For peer review only - http://bmjopen.bmj.com/site/about/guidelines.xhtml

For peer review only

For peer review only - http://bmjopen.bmj.com/site/about/guidelines.xhtml

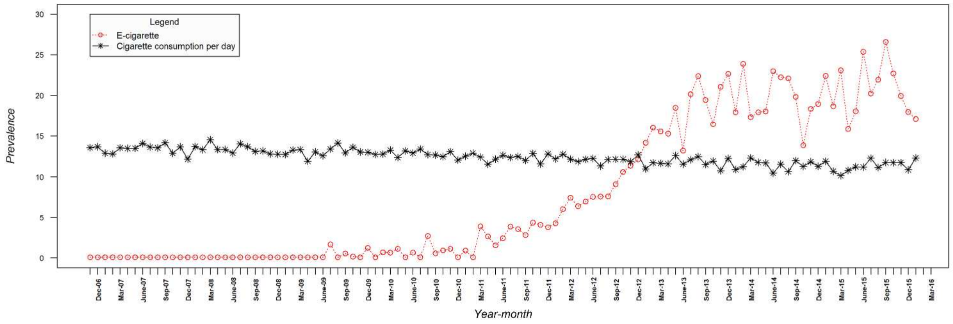

Figure 1: Monthly prevalence of cigarette consumption and e-cigarette use for harm reduction among smokers

381x177mm (300 x 300 DPI)

er review only

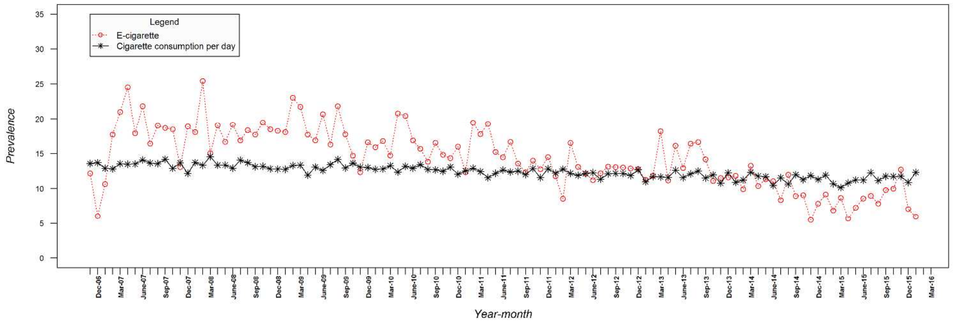

Figure 2: Monthly prevalence of cigarette consumption and NRT use for harm reduction among smokers

381x177mm (300 x 300 DPI)

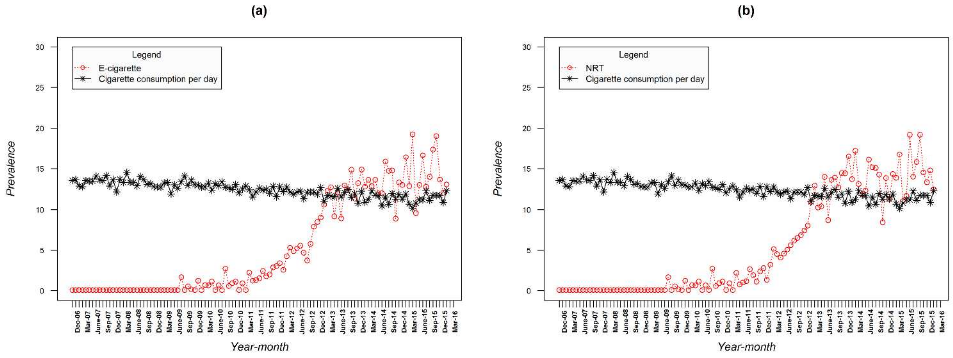

Supplementary Figure 1: Monthly prevalence of cigarette consumption and e-cigarette use for a) cutting down and b) temporary abstinence among smokers

381x177mm (300 x 300 DPI)

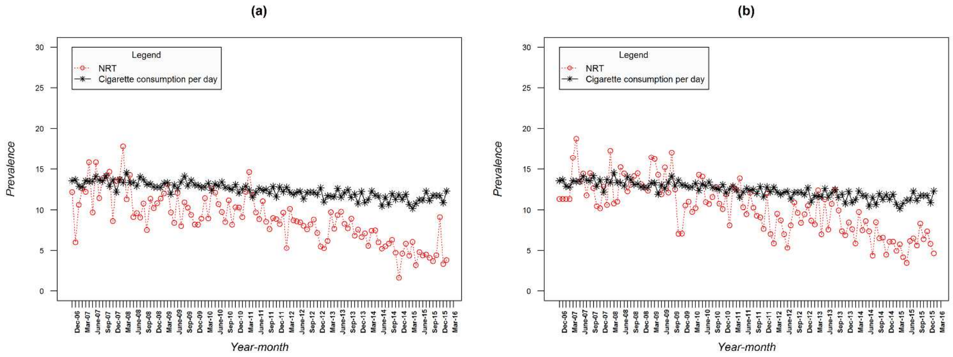

Supplementary Figure 2: Monthly prevalence of cigarette consumption and NRT use for a) cutting down and b) temporary abstinence among smokers

381x177mm (300 x 300 DPI)

For peer review only

STROBE 2007 (v4) Statement—Checklist of items that should be included in reports of *cross-sectional studies*

Section/Topic	Item #	Recommendation	Reported on page #
**Title and abstract**	1	(*a*) Indicate the study's design with a commonly used term in the title or the abstract	1
		(*b*) Provide in the abstract an informative and balanced summary of what was done and what was found	2
**Introduction**			
Background/rationale	2	Explain the scientific background and rationale for the investigation being reported	3
Objectives	3	State specific objectives, including any prespecified hypotheses	3
**Methods**			
Study design	4	Present key elements of study design early in the paper	4/5
Setting	5	Describe the setting, locations, and relevant dates, including periods of recruitment, exposure, follow-up, and data collection	4/5
Participants	6	(*a*) Give the eligibility criteria, and the sources and methods of selection of participants	4/5
Variables	7	Clearly define all outcomes, exposures, predictors, potential confounders, and effect modifiers. Give diagnostic criteria, if applicable	4/5
Data sources/ measurement	8*	For each variable of interest, give sources of data and details of methods of assessment (measurement). Describe comparability of assessment methods if there is more than one group	4/5
Bias	9	Describe any efforts to address potential sources of bias	*5/6*
Study size	10	Explain how the study size was arrived at	6
Quantitative variables	11	Explain how quantitative variables were handled in the analyses. If applicable, describe which groupings were chosen and why	5/6
Statistical methods	12	(*a*) Describe all statistical methods, including those used to control for confounding	5/6
		(*b*) Describe any methods used to examine subgroups and interactions	5/6
		(*c*) Explain how missing data were addressed	5/6
		(*d*) If applicable, describe analytical methods taking account of sampling strategy	5./6
		(*e*) Describe any sensitivity analyses	5/6
**Results**			

For peer review only

Participants	13*	(a) Report numbers of individuals at each stage of study—eg numbers potentially eligible, examined for eligibility, confirmed eligible, included in the study, completing follow-up, and analysed	6
		(b) Give reasons for non-participation at each stage	N/A
N/A		(c) Consider use of a flow diagram	
Descriptive data	14*	(a) Give characteristics of study participants (eg demographic, clinical, social) and information on exposures and potential confounders	6
		(b) Indicate number of participants with missing data for each variable of interest	N/A
Outcome data	15*	Report numbers of outcome events or summary measures	6/7
Main results	16	(*a*) Give unadjusted estimates and, if applicable, confounder-adjusted estimates and their precision (eg, 95% confidence interval). Make clear which confounders were adjusted for and why they were included	6/7
		(*b*) Report category boundaries when continuous variables were categorized	N/A
		(*c*) If relevant, consider translating estimates of relative risk into absolute risk for a meaningful time period	N/A
Other analyses	17	Report other analyses done—eg analyses of subgroups and interactions, and sensitivity analyses	6/7
**Discussion**			
Key results	18	Summarise key results with reference to study objectives	7/8
Limitations	19	Discuss limitations of the study, taking into account sources of potential bias or imprecision. Discuss both direction and magnitude of any potential bias	7/8
Interpretation	20	Give a cautious overall interpretation of results considering objectives, limitations, multiplicity of analyses, results from similar studies, and other relevant evidence	7/8
Generalisability	21	Discuss the generalisability (external validity) of the study results	7/8
**Other information**			
Funding	22	Give the source of funding and the role of the funders for the present study and, if applicable, for the original study on which the present article is based	8

*Give information separately for cases and controls in case-control studies and, if applicable, for exposed and unexposed groups in cohort and cross-sectional studies.

Note: An Explanation and Elaboration article discusses each checklist item and gives methodological background and published examples of transparent reporting. The STROBE checklist is best used in conjunction with this article (freely available on the Web sites of PLoS Medicine at http://www.plosmedicine.org/, Annals of Internal Medicine at http://www.annals.org/, and Epidemiology at http://www.epidem.com/). Information on the STROBE Initiative is available at www.strobe-statement.org.

For peer review only

Is prevalence of e-cigarette and nicotine replacement therapy use among smokers associated with average cigarette consumption in England? A time-series analysis

Journal:	*BMJ Open*
Manuscript ID	bmjopen-2017-016046.R1
Article Type:	Research
Date Submitted by the Author:	21-Jun-2017

Complete List of Authors:	Beard, Emma; UCL,<br>Brown, Jamie; University College London, Psychology & Language Sciences<br>Michie, Susan; University College London, Centre for Outcomes Research and Effectivenes<br>West, Robert; University College London, Epidemiology and Public Health

**Primary Subject Heading**:	Smoking and tobacco
Secondary Subject Heading:	Addiction
Keywords:	Time series, ARIMAX, SMOKING, E-CIGARETTE, NRT

SCHOLARONE™ Manuscripts

Is prevalence of e-cigarette and nicotine replacement therapy use among smokers associated with average cigarette consumption in England? A time-series analysis

Emma Beard[1,2], Jamie Brown[1,2], Susan Michie[2] & Robert West[1]

1 Cancer Research UK Health Behaviour Research Centre, University College London, WC1E 6BT, UK

2 Research Department of Educational, Clinical and Health Psychology, University College London, WC1E 7HB

For peer review only

Word count: 2843

Journal: BMJ Open

Correspondence to: Emma Beard, Cancer Research UK Health Behaviour Research Centre, University College London, WC1E 6BP. Email: e.beard@ucl.ac.uk. Tel: 02031083179

For peer review only - http://bmjopen.bmj.com/site/about/guidelines.xhtml

ABSTRACT

Objectives: Many smokers use e-cigarettes and licensed nicotine replacement therapy (NRT), often in an attempt to reduce their cigarette consumption. We estimated how far changes in prevalence of e-cigarette and NRT use while smoking were accompanied by changes in cigarette consumption at the population level.

Design: Repeated representative cross-sectional population surveys of adults aged 16+ in England

Methods: We used ARIMAX modelling of monthly data between 2006 and 2016 from the Smoking Toolkit Study. Prevalence of e-cigarette use and NRT use in current smokers, and specifically for smoking reduction and temporary abstinence, were input variables. Mean daily cigarette consumption was the dependent variable. Analyses involved adjustment for mass media expenditure and tobacco control policies.

Results: No statistically significant associations were found between changes in use of e-cigarettes (β -0.012, 95%CI -0.026 to 0.002) or NRT (β 0.015 95%CI -0.026 to 0.055) while smoking and daily cigarette consumption. Neither did we find clear evidence for an association between e-cigarette use (β -0.010 95%CI -0.025 to 0.005 and β 0.011 95% -0.027 to 0.004) or NRT use (β 0.006 95% -0.030 to 0.043 and β 0.022 95% -0.020 to 0.063) specifically for smoking reduction and temporary abstinence respectively, and changes in daily cigarette consumption.

Conclusion: If use of e-cigarettes and licensed nicotine replacement therapy while smoking acted to reduce cigarette consumption in England between 2006 and 2016, the effect was likely very small at a population level.

Strengths and limitations of this study

- This is the first time series study to assess the population level impact of the use of NRT and e-cigarettes for harm reduction on cigarette consumption
- This study uses a large representative sample of the population in England and considers both smoking reduction and temporary abstinence
- A wide range of confounders are adjusted for including population level interventions
- In countries with weaker tobacco control, or stricter regulation of using products for harm reduction, different effects may be observed.
- Data are observational and so strong conclusions regarding cause and effect cannot be made

For peer review only - http://bmjopen.bmj.com/site/about/guidelines.xhtml

INTRODUCTION

Randomised controlled trials have shown that use of non-tobacco nicotine containing products (e.g. Nicotine Replacement Therapy (NRT)) are efficacious for harm reduction attempts [1]. Harm reduction is defined as any attempt to reduce the harm from smoking without an intention to quit completely, such as, the use of NRT for smoking reduction (i.e. during attempts to cut down) or during periods of temporary abstinence (i.e. during periods of time when one is unable to smoke) [1]. Outside of the clinical setting where little behavioural support is provided, the use of NRT during attempts to cut down appears to increase smoker's propensity to quit, but does not result in significantly large reductions in cigarette consumption [2-4]. Explanations for this include the lack of behavioural support and possible poor compliance with the medical regimen [5 6].

In recent years, there has been an increase in the overall use of nicotine containing products for harm reduction, with a growth in e-cigarettes more than offsetting a decline in the use of NRT [7-9]. Previous studies suggest that e-cigarettes which contain nicotine reduce cravings more effectively than NRT [7 10 11], have better adherence rates [7 12], and deliver clinically significant levels of nicotine into the blood, at least for some smokers [10 11 13]. Thus, although further studies are needed it is possible that e-cigarettes may be a more effective aid for smoking reduction than licensed nicotine products [14 15]. However, it also remains possible that e-cigarettes will not result in clinically significant reductions in cigarette intake at a population level.

The aim of this study was to assess the association between changes in prevalence of e-cigarettes and NRT with changes in mean cigarette consumption per day using a time-series approach. Time-series analysis allows us to take into account underlying trends, the effect of other tobacco control interventions, autocorrelation (whereby data collected at points closer in time tends to be more similar), and to consider possible lag effects of the independent variable on the dependent variable [16]. Where associations are found, they cannot unequivocally establish a causal association but can be indicative, as has been the case with estimating the effect of price of cigarettes on population consumption [17], mass media expenditure on use of specialist stop smoking services [18], and introduction of varenicline to the market on prevalence of use of smoking cessation medication [19]. Where associations are not found, or they go in a direction opposite to that expected, this can also be informative.

Specifically, this paper assesses the association between mean cigarette consumption per day with:

1. Current e-cigarette use among smokers for any purpose, current use specifically for smoking reduction, and current use specifically for temporary abstinence
2. Current NRT use among smokers for any purpose, current use specifically for smoking reduction, and current use specifically for temporary abstinence

Sensitivity analyses will examine the effect of focusing only on daily e-cigarette and NRT use, given previous associations between extent of non-tobacco nicotine containing product use and the effectiveness of harm reduction attempts [6].

METHODS

Design

We used ARIMAX modelling of monthly data between 2006 and 2016 primarily from the Smoking Toolkit Study. The STS is a monthly survey of a representative sample of the population in England aged 16+ [20]. This has been collecting data on smoking patterns among smokers and recent ex-smokers since November 2006. Questions on the use of e-cigarettes among all smokers were

For peer review only - http://bmjopen.bmj.com/site/about/guidelines.xhtml

introduced in May 2011 and as aids to a quit attempt among smokers attempting to stop in July 2009. The STS involves monthly household surveys using a random location sampling design, with initial random selection of grouped output areas (containing 300 households), stratified by ACORN (sociodemographic) characteristics (http://www.caci.co.uk/acron/acornmap.asp) and region. Interviewers then choose which houses within these areas are most likely to fulfil quotas based on the probability of being at home tailored to region and conduct face-to-face computer assisted interviews with one member per household. Participants from the STS appear to be representative of the population in England, having similar socio-demographic composition as other large national surveys, such as the Health Survey for England [20].

Measures

Explanatory variables

Daily and non-daily smokers were asked the following questions:

1. Which, if any, of the following are you currently using to help you cut down the amount you smoke?
2. Do you regularly use any of the following in situations when you are not allowed to smoke?
3. Can I check, are you using any of the following either to help you stop smoking, to help you cut down or for any other reason at all?

All three questions had the following responses options: nicotine gum, nicotine replacement lozenges\tablets, nicotine replacement inhaler, nicotine replacement nasal spray, nicotine patch, electronic cigarette, nicotine mouth spray, other, none.

Current e-cigarette use was derived by an 'electronic cigarette' response to any of the three questions; e-cigarette use for smoking reduction by a response to the first question; and e-cigarette use for temporary abstinence by a response to the second question.

Current NRT use was derived by a NRT product response ('nicotine gum, nicotine replacement lozenges\tablets, nicotine replacement inhaler, nicotine replacement nasal spray, nicotine patch, or nicotine mouth spray') to any of the three questions; NRT use for smoking reduction by a NRT product response to the first question; and NRT use for temporary abstinence by a NRT product response to the second question.

An 'electronic cigarette' response E-cigarettes: current use Current e-cigarette use was derived by smokers answering electronic cigarettes to any of the Smokers answering *electronic* cigarettes to any of the three questions were categorised as current e-cigarette users, while smokers answering an *NRT* product were classed as current NRT users. Responses to the first question were used to derive prevalence of e-cigarette use for smoking reduction and NRT use for smoking reduction, while responses to the second question were used to derive prevalence of e-cigarette for temporary abstinence and NRT use for temporary abstinence. Data were not recorded on NRT use for temporary abstinence between Nov-2006 and Jan-2007 and was imputed using prevalence data from Feb-2007.

Data were only available on the prevalence of use of electronic cigarettes among smokers from April 2011 – although use specifically during a recent quit attempt were available from July 2009. Thus, prevalence of electronic cigarette use among smokers between July 2009 and April 2011 was estimated from data on use during a quit attempt; use of electronic cigarettes among smokers between November 2006 and June 2009 was assumed to be 0.1% of smokers based on other surveys, which found their use to be very rare before 2009 [21 22].

For peer review only - http://bmjopen.bmj.com/site/about/guidelines.xhtml

Daily NRT and e-cigarette users were classified as those who reported that they used the product(s) at least once per day in response to the question: How many times per day on average do you use your nicotine replacement product or products? This question was introduced in July-2010. Prior to this time, prevalence of daily NRT use was assumed to be 60% of all users [6], while e-cigarette prevalence was computed as above using prevalence during a quit attempt or 0.1%.

Outcome variables

Smokers taking part in the STS were also asked how many cigarettes they smoke on average per day. Non-daily smokers were asked how many cigarettes they smoked per week, which was then converted to a daily figure.

Co-variables

In England, tobacco mass media campaigns have been run as part of a national tobacco control programme. Spending was almost completely suspended in 2010 and then re-introduced in 2011 at a much lower level. Previous studies have shown that such cuts were associated with a decreased use of smoking cessation support [18 23]. Thus, advertising expenditure will be adjusted for using data obtained from Public Health England. Data on mass media expenditure was available monthly from May 2008, and yearly prior to this period, and so a monthly average was assumed. For a number of months spending was effectively zero and was imputed as 0.1 to allow the analysis to run.

A number of tobacco control policies were adjusted for. These included the move in commissioning of stop smoking services to local authorities in April 2013 [24], introduction of a smoking ban in July 2007 [25], licensing of NRT for harm reduction in December 2009 [26], the publication of NICE guidance on harm reduction in June 2013 [27], and change in the minimum age of sale of cigarettes October 2007 [28]. Price of cigarettes is correlated 0.99 with time and will thereby be taken into account by use of differencing (i.e. using the differences between consecutive observation rather the observations themselves) to make the series stationary.

Ethical approval:

Ethical approval for the Smoking Toolkit Study was granted by the UCL ethics committee (ID 0498/001).

Analysis

The analysis plan was registered on the Open Science Framework prior to data analysis (https://osf.io/6swk3/). All data were analysed in R version 3.2.4 [29] using Autoregressive Integrated Moving Average with Exogeneous Input (ARIMAX) modelling [16 30 31]. Data were weighted prior to the analyse to match the population in England using a rim (marginal) weighting technique. This involves an iterative sequence of weighting adjustments whereby separate nationally representative target profiles are set (for gender, working status, children in the household, age, social-grade and region). This process is then repeated until all variables match the specified targets (for more details see [20]).

Two waves of data were collected in March 2007 and March 2013, these waves were averaged. No data were collected in December 2008. Mean cigarette consumption, NRT use and e-cigarette use during this period was calculated as an average of the month before and the month after. For a few months (May-2012, Jul-2012, Sep-2012, Nov-2012, Jan-2013, Mar-2013), data on electronic cigarettes and NRT use among smokers were not recorded. For these months, the average of the previous and next month was imputed.

For peer review only - http://bmjopen.bmj.com/site/about/guidelines.xhtml

The Granger causality test suggested that there was some evidence for the violation of the assumption of weak exogeneity (i.e. Y can depend on the lagged values of X but the reverse must not be true) between the input and the output series. However, caution has been advised when using this and similar tests on data across a long time-series [32 33] and there was no theoretical reason we could identify for a bi-directional relationship between e-cigarette use and cigarette consumption. It was assumed that the association was spurious and likely removed following adjustment for other covariates.

Both unadjusted and fully adjusted models are reported which regressed onto mean cigarette consumption per day 1) use of e- cigarettes among current smokers; 2) use of e- cigarettes for smoking reduction; 3) use of e- cigarettes for temporary abstinence; 4) use of NRT for harm reduction; 5) use of NRT for temporary abstinence and 6) use of NRT for smoking reduction. Sensitivity analyses were conducted which constrained the analysis to only those reporting daily e-cigarette and NRT use. We followed a standard ARIMAX modelling approach [16 34]. The series were first log-transformed to stabilise the variance, and if required, first differenced and seasonally differenced. The autocorrelation and partial autocorrelation functions were then examined in order to determine the seasonal and non-seasonal moving average (MA) and autoregressive terms (AR). For example, AR(1) means that the value of a series at one point in time is the sum of a fraction of the value of the series at the immediately preceding point in time and an error component; while MA(1) means that the value of a series at one point in time is a function of a fraction of the error component of the series at the immediately preceding point in time and an error component at the current point in time. To identify the most appropriate transfer function (i.e. lag) for the continuous explanatory variables the sample cross-correlation function was checked for each ARIMAX model. Coefficients can be interpreted as estimates of the percentage change in cigarette consumption for every a) percentage increase in use of e-cigarettes and NRT, b) percentage increase in mass media expenditure, and c) implementation of tobacco control policies.

Bayes factors (BF) were derived for non-significant findings using an online calculator [35] to disentangle whether there is evidence for the null hypothesis of no effect (Bayes Factor <1/3rd) or the data are insensitive (Bayes Factor between 1/3rd and 3). A half-normal distribution was assumed with a percentage change in the outcomes of interest for every percentage increase in the input series of 0.009% based on the effect detectable with 80% power (see sample size). Sensitivity analyses were conducted using a much larger percentage change of 0.1. This was based on a meta-analysis assessing the efficacy of non-tobacco nicotine replacement products for harm reduction, which reported that 21.8% of the experimental group had reduced consumption by more than 50% at final follow-up compared with 16.5% receiving placebo [1]. We therefore assumed that a 5% change in prevalence of NRT and e-cigarettes would be associated with a 0.5% change in overall cigarette consumption.

STROBE guidelines for the reporting of observational studies were followed throughout [36].

Sample size

Simulation-based power analyses suggested that this study would have 80% power to detect a change in the output series of 0.009% for every 1% change in the input series, assuming 113 monthly data collection points, MA(1) autocorrelation [37], a baseline proportion for the input series of 0.005 [9], a baseline mean (SD) for the output series of 12.3 [38], and a total change over time for the input series of 30% [38].

RESULTS

Sample characteristics

For peer review only - http://bmjopen.bmj.com/site/about/guidelines.xhtml

Data were collected on 199,483 adults aged 16+ taking part in the STS who reported their smoking status between November 2006 and March 2016. Of these, 43,608 (20.8%: 95%CI 20.6 to 21.0) were current smokers. Fifty-two percent (95%CI 52.0 to 53.0) of smokers were male and 60.4% (95%CI 60.0 to 60.1%) were in routine or manual positions or were unemployed. The average age of smokers in this study was 42.1 years (95%CI 42.0 to 42.1)

Main analysis

Figure 1 shows that cigarette consumption declined over the study period from 13.6 to 12.3 (mean 12.4, SD 0.92). This figure also shows that current use of e-cigarettes among smokers for harm reduction increased from negligible use in the last quarter of 2006 to 17.1% at the end of the study (mean 7.8%, SD 8.82). Figure 2 shows that there was also a decline in the use of NRT for harm reduction from 12.2% to 6.0% (mean 14.4%, SD 4.36). Supplementary Figures 1 and 2 show the changes in e-cigarette and NRT use for smoking reduction and temporary abstinence, respectively.

Tables 1, 2 and 3 show the results of the ARIMAX models assessing the association between cigarette consumption per day with 1) e- cigarette use among current smokers and NRT use for harm reduction; 2) e- cigarette and NRT use for smoking reduction and 3) e- cigarette and NRT use for temporary abstinence. The findings were inconclusive as to whether or not an association was present between use of e-cigarettes and NRT for any purpose and cigarette consumption.

Bayes Factors were between 1/3rd and 3 when assuming a 0.009% change in cigarette consumption for every percentage change in the input series, suggesting the data are insensitive to detect very small reductions in cigarette consumption. Most Bayes Factors were <1/3rd, when assuming a 0.1% change in cigarette consumption for every percentage change in the input series, suggesting evidence for the null hypothesis that NRT use and e-cigarette use among smokers has not resulted in large reductions in cigarette intake.

For peer review only - http://bmjopen.bmj.com/site/about/guidelines.xhtml

Sensitivity analysis

Current daily use of e-cigarettes among smokers for harm reduction increased from negligible use in the last quarter of 2006 to 11.1% at the end of the study (mean 4.5%, SD 4.91). There was also an increase in e-cigarette use specifically for temporary abstinence (from 0.1% to 8.4%; mean 3.5% SD 3.81) and smoking reduction (from 0.1% to 8.3%; mean 3.3% SD 3.64).

In contrast, there was a decline in the use of NRT for harm reduction from 7.3% to 2.9% (mean 6.5%, SD 2.35) and a decline in NRT use specifically for temporary abstinence (from 7.3% to 1.8%; mean 4.7% SD 2.29) and smoking reduction (from 6.8% to 2.6%; 5.8% SD 2.46).

Tables 1, 2 and 3 also show the results of the sensitivity analyses restricted to those smokers using NRT or e-cigarettes daily. The findings were inconclusive as to whether or not an association was present between the daily use of e- cigarettes and NRT for any purpose and cigarette consumption. Bayes Factors suggested the data are insensitive to detect very small reductions in cigarette consumption, but there is evidence for the null hypothesis that NRT use and e-cigarette use among smokers has not resulted in large reductions in cigarette intake.

DISCUSSION

To our knowledge, this is the first empirical study to estimate the population association between the use of e-cigarettes and NRT among current smokers on cigarette consumption per day, using a time-series approach. There was evidence that there was no substantial association between the rise in use of e-cigarettes and decline in NRT use and changes in cigarette consumption per day.

Strengths and limitations

A strength of the study is the use of a large representative sample of the population in England, stratification of results by daily use, and the consideration of both temporary abstinence and smoking reduction. Previous studies have shown that reductions in cigarette intake are dependent on the extent of NRT use and differ as a function of the specific harm reduction behaviour i.e. an attempt to cut down or restraining from smoking during periods of brief abstinence [2 6].

The study had a number of limitations. First, caution should be taken when interpreting estimates of the covariates, i.e. impact of some of the tobacco control policies, as interrupted explanatory variables with short time-periods prior to their introduction in ARIMA type models often give inaccurate estimates of the standard errors [28]. Thus, although the increase in age-of-sale has been previously associated with a decline in smoking prevalence [24], the short lead in period may have masked any true association [27]. Secondly, the STS required participants to recall their average daily cigarette intake which is likely to have been somewhat inaccurate. Thirdly, the findings may not generalise to other countries. England has a strong tobacco control climate and relatively liberal attitude towards harm reduction and e-cigarette use. In countries with weaker tobacco control, or stricter regulation of using products for harm reduction, different effects may be observed. Fourthly, although we are unaware of any other major population level interventions or other events during the study period, we cannot rule out residual confounding. Fifthly, participants were not asked questions regarding potentially important features of the e-cigarette (e.g., nicotine content, flavouring, device type) or frequency and duration of use. It is likely that these factors may play a role in their effectiveness and should be considered in future studies [15 39]. Finally, as data were not collected on current e-cigarette use prior to April 2011, prevalence was estimated from use during a quit attempt or from previous studies [21 22]. This was necessary to ensure that the time series was long enough for an ARIMAX analysis and is an appropriate approach when data are

For peer review only - http://bmjopen.bmj.com/site/about/guidelines.xhtml

missing completely at random [16 40]. As prevalence was low and relatively stable during this period it is unlikely to have impacted on the reported results.

Implications of findings

The findings are in line with previous studies which show that reductions in cigarette consumption observed in clinical trials of NRT for harm reduction do not appear to generalise beyond the closely controlled trial setting [1 2]. It was hypothesised that e-cigarettes may be associated with population mean cigarette intake given that they reduce cravings more effectively than NRT [7 10 11], have better adherence rates [7 12], and deliver clinically significant levels of nicotine into the blood [10 11 13][11].

The finding that e-cigarette use was not associated with reductions in consumption at a population level is consistent with previous real-world studies at the individual level. These have found little change in consumption among ever e-cigarette users [41] and that only a minority of daily users manage to reduce by a substantial amount which is not likely to be detected at a population level [42]. The findings of a recent pragmatic controlled trial, whereby 60% of participants using e-cigarettes had managed to reduce by over 50% by 6 months follow-up, suggests that the lack of effectiveness at a population level may not be the consequence of poor behavioural support [11]. .

Of course, it remains plausible that e-cigarettes may still be associated with a small effect on mean population cigarette consumption [15], and that a reduction in harm from smoking at a population level could be seen through their promotion of quit attempts [37] or by reducing smoke intake from each cigarette [5].

Conclusion

In conclusion, the increased prevalence of e-cigarettes use among smokers in England has not been associated with a detectable change in cigarette consumption per day. The decline in the use of NRT has also not been associated with a change in mean cigarette intake. If use of e-cigarettes and licensed nicotine replacement therapy while smoking acts to reduce cigarette consumption, the effect is probably small.

Declaration of interests

RW undertakes consultancy and research for and receives travel funds and hospitality from manufacturers of smoking cessation medications but does not, and will not take funds from e-cigarettes manufacturers or the tobacco industry. RW and SM are honorary co-directors of the National Centre for Smoking Cessation and Training. RW is a Trustee of the stop-smoking charity, QUIT. RW salary is funded by Cancer Research UK. SM salary is funded by Cancer Research UK and by the National Institute for Health Research (NIHR)'s School for Public Health Research (SPHR). EB and JB have received unrestricted research funding from Pfizer. EB and JB are funded by CRUK. EB is also funded by NIHR's SPHR and JB by the Society for the Study of Addiction. RW has received travel funds and hospitality from, and undertaken research and consultancy for pharmaceutical companies that manufacture or research products aimed at helping smokers to stop. These products include nicotine replacement therapies, Champix (varenicline) and Zyban (bupropion). This has led to payments to him personally and to his institution.

Author contributions

EB, JB, SM and RW designed the study. EB wrote the first draft and conducted the analyses. All authors commented on this draft and contributed to the final version.

Data sharing

For peer review only - http://bmjopen.bmj.com/site/about/guidelines.xhtml

For access to the data please contact the lead author, Dr Emma Beard e.beard@ucl.ac.uk

Sources of funding

The Smoking Toolkit Study is currently primarily funded by Cancer Research UK (C1417/A14135; C36048/A11654; C44576/A19501) and has previously also been funded by Pfizer, GSK, and the Department of Health. JB's post is funded by a fellowship from the Society for the Study of Addiction and CRUK also provide support (C1417/A14135); RW is funded by Cancer Research UK (C1417/A14135); EB is funded by a fellowship from the NIHR SPHR (SPHR-SWP-ALC-WP5) and CRUK also provide support (C1417/A14135). SW is funded by Cancer Research UK (C1417/A14135) and NIHR SPHR (SPHR-SWP-ALC-WP5) also provide support. SPHR is a partnership between the Universities of Sheffield; Bristol; Cambridge; Exeter; UCL; The London School for Hygiene and Tropical Medicine; the LiLaC collaboration between the Universities of Liverpool and Lancaster and Fuse; The Centre for Translational Research in Public Health, a collaboration between Newcastle, Durham, Northumbria, Sunderland and Teesside Universities. The views expressed are those of the authors(s) and not necessarily those of the NHS, NIHR, or Department of Health. No funders had any involvement in the design of the study, the analysis or interpretation of the data, the writing of the report, or the decision to submit the paper for publication

Figure 1: Monthly prevalence of cigarette consumption and e-cigarettes for harm reduction among smokers

Figure 2: Monthly prevalence of cigarette consumption and NRT use for harm reduction among smokers

Supplementary ure 1: Monthly prevalence of cigarette consumption and e-cigarette use for a) cutting down and b) temporary abstinence among smokers

Supplementary Figure 2 Monthly prevalence of cigarette consumption and NRT use for a) cutting down and b) temporary abstinence among smokers

For peer review only

Table 1: Estimated percentage point changes in mean cigarette consumption per day as a function of e- cigarette use and NRT use among smokers from November 2006 until March 2016, based on Autoregressive Integrated Moving Average with Exogeneous Input (ARIMAX) models

	**All users of nicotine replacement**			**Only daily users of nicotine replacement**		
	Percentage change per 1% change in the exposure (95%CI)<br>p	Percentage change per 1% change in the exposure (95%CI)<br>p	Percentage change per 1% change in the exposure (95%CI)<br>P	Percentage change per 1% change in the exposure (95%CI)<br>p	Percentage change per 1% change in the exposure (95%CI)<br>p	Percentage change per 1% change in the exposure (95%CI)<br>p
Any current use of e- cigarettes<br>(Immediate impact)	-0.011 (-0.025 to 0.002)<br>0.097		-0.012 (-0.026 to 0.002)<br>0.091	-0.010 (-0.024 to 0.004)<br>0.149		-0.011 (-0.026 to 0.003)<br>0.130
NRT use for harm reduction<br>(Immediate impact)		0.012 (-0.028 to 0.053)<br>0.546	0.015 (-0.026 to 0.055)<br>0.475		0.003 (-0.019 to 0.025)<br>0.794	0.005 (-0.017 to 0.027)<br>0.672
Mass media expenditure<br>(Immediate impact)			<0.001 (-0.001 to 0.001)<br>0.984			<0.001(-0.001 to 0.001)<br>0.880
	Total percentage change due to the exposure (95%CI)<br>p	Total percentage change due to the exposure (95%CI)<br>p	Total percentage change due to the exposure (95%CI)<br>P	Total percentage change due to the exposure (95%CI)<br>p	Total percentage change due to the exposure (95%CI)<br>P	Total percentage change due to the exposure (95%CI)<br>P
Smoking ban<br>(Pulse effect)			0.015 (-0.070 to 0.101)<br>0.724			0.013 (-0.072 to 0.099)<br>0.756
Increase in age-of-sale<br>(Pulse effect)			-0.041 (-0.126 to 0.044)<br>0.342			-0.043 (-0.128 to 0.042)<br>0.324
Move to local authority control<br>(Pulse effect)			-0.019 (-0.105 to 0.067)<br>0.662			-0.027 (-0.112 to 0.058)<br>0.533
Licensing for NRT for harm reduction<br>(Pulse effect)			0.021 (-0.067 to 0.110)<br>0.639			0.020 (-0.069 to 0.109)<br>0.661
NICE guidance on harm reduction<br>(Pulse effect)			-0.024 (-0.109 to 0.061)<br>0.578			-0.028 (-0.114 to 0.057)<br>0.512
Best fitting model	ARIMA(0,1,1)(0,0,0)^{12}	ARIMA(0,1,1)(0,0,0)^{12}	ARIMA(0,1,1)(0,0,0)^{12}	ARIMA(0,1,1)(0,0,0)^{12}	ARIMA(0,1,1)(0,0,0)^{12}	ARIMA(0,1,1)(0,0,0)^{12}
Non-seasonal AR *p*	NA	NA	NA	NA	NA	NA
Non-seasonal MA *p*	<0.001	<0.001	<0.001	<0.001	<0.001	<0.001
Seasonal AR *p*	NA	NA	NA	NA	NA	NA
Seasonal MA *p*	NA	NA	NA	NA	NA	NA
R-squared	0.65	0.65	0.66	0.65	0.64	0.66
**Bayes Factor e-cigarette [0.009(0.1)]**	2.44 (0.46)		2.68 (0.55)	1.95 (0.35)		2.12 (0.41)
**Bayes Factor NRT [0.009(0.1)]**		0.77 (0.14)	0.74 (0.13)		0.69 (0.09)	0.63 (0.08)

Note: 95%CI=95% confidence interval; MA=moving average; AR=autoregressive; NA=not applicable; An AR(1) means that the value of a series at one point in time is the sum of a fraction of the value of the series at the immediately preceding point in time and an error component; An MA(1) means that the value of a series at one point in time is a function of a fraction of the error component of the series at the immediately preceding point in time and an error component at the current point in time.

For peer review only - http://bmjopen.bmj.com/site/about/guidelines.xhtml

Table 2: Estimated percentage point changes in mean cigarette consumption per day as a function of e- cigarette use and NRT use among smokers for cutting down from November 2006 until March 2016, based on Autoregressive Integrated Moving Average with Exogeneous Input (ARIMAX) models

	**All users of nicotine replacement**			**Only daily users of nicotine replacement**		
	Percentage change per 1% change in the exposure (95%CI) P	Percentage change per 1% change in the exposure (95%CI) p	Percentage change per 1% change in the exposure (95%CI) P	Percentage change per 1% change in the exposure (95%CI) P	Percentage change per 1% change in the exposure (95%CI) P	Percentage change per 1% change in the exposure (95%CI) P
Use of e- cigarettes for cutting down (Immediate impact)	-0.010 (-0.024 to 0.005) 0.191		-0.010 (-0.025 to 0.005) 0.191	-0.008 (-0.023 to 0.006) 0.256		-0.009 (-0.024 to 0.006) 0.229
NRT use for cutting down (Immediate impact)		0.002 (-0.033 to 0.037) 0.917	0.006 (-0.030 to 0.043) 0.732		-0.002 (-0.016 to 0.013) 0.825	-0.002 (-0.017 to 0.013) 0.786
Mass media expenditure (Immediate impact)			<0.001 (-0.001 to 0.001) 0.885			<0.001 (-0.001 to 0.001) 0.860
	Total percentage change due to the exposure (95%CI) P	Total percentage change due to the exposure (95%CI) p	Total percentage change due to the exposure (95%CI) P	Total percentage change due to the exposure (95%CI) P	Total percentage change due to the exposure (95%CI) P	Total percentage change due to the exposure (95%CI) P
Smoking ban (Pulse effect)			0.014 (-0.072 to 0.099) 0.755			0.012 (-0.073 to 0.097) 0.782
Increase in age-of-sale (Pulse effect)			-0.043 (-0.128 to 0.042) 0.323			-0.042 (-0.127 to 0.043) 0.329
Move to local authority control (Pulse effect)			-0.025 (-0.110 to 0.061) 0.571			-0.029 (-0.115 to 0.056) 0.499
Licensing for NRT for harm reduction (Pulse effect)			0.018 (-0.072 to 0.108) 0.694			0.015 (-0.074 to 0.103) 0.747
NICE guidance on harm reduction (Pulse effect)			-0.028 (0.058 to <0.001) 0.529			-0.027 (-0.112 to 0.059) 0.541
Best fitting model	ARIMA(0,1,1)(0,0,0)^{12}	ARIMA(0,1,1)(0,0,0)^{12}	ARIMA(0,1,1)(0,0,0)^{12}	ARIMA(0,1,1)(0,0,0)^{12}	ARIMA(0,1,1)(0,0,0)^{12}	ARIMA(0,1,1)(0,0,0)^{12}
Non-seasonal AR *p*	NA	NA	NA	NA	NA	NA
Non-seasonal MA *p*	<0.001	<0.001	<0.001	<0.001	<0.001	<0.001
Seasonal AR *p*	NA	NA	NA	NA	NA	NA
Seasonal MA *p*	NA	NA	NA	NA	NA	NA
R-squared	0.64	0.64	0.65	0.64	0.64	0.65
**Bayes Factor e-cigarette [0.009(0.1)]**	1.87 (0.34)		1.79 (0.32)	1.46 (0.23)		1.61 (0.27)
**Bayes Factor NRT [0.009(0.1)]**		0.86 (0.16)	0.81 (0.15)		0.76 (0.10)	0.76 (0.10)

Note: 95%CI=95% confidence interval; MA=moving average; AR=autoregressive; NA=not applicable; An AR(1) means that the value of a series at one point in time is the sum of a fraction of the value of the series at the immediately preceding point in time and an error component; An MA(1) means that the value of a series at one point in time is a function of a fraction of the error component of the series at the immediately preceding point in time and an error component at the current point in time.

For peer review only - http://bmjopen.bmj.com/site/about/guidelines.xhtml

Table 3: Estimated percentage point changes in mean cigarette consumption per day as a function of e- cigarette use and NRT use among smokers for temporary abstinence from November 2006 until March 2016, based on Autoregressive Integrated Moving Average with Exogeneous Input (ARIMAX) models

	**All users of nicotine replacement**			**Only daily users of nicotine replacement**		
	Percentage change per 1% change in the exposure (95%CI) P	Percentage change per 1% change in the exposure (95%CI) p	Percentage change per 1% change in the exposure (95%CI) P	Percentage change per 1% change in the exposure (95%CI) p	Percentage change per 1% change in the exposure (95%CI) p	Percentage change per 1% change in the exposure (95%CI) P
Use of e- cigarettes for temporary abstinence (Immediate impact)	-0.010 (-0.024 to 0.005) 0.150		-0.011 (-0.027 to 0.004) 0.146	-0.010 (-0.024 to 0.004) 0.159		-0.011 (-0.026 to 0.003) 0.135
NRT use for temporary abstinence (Immediate impact)		0.023 (-0.016 to 0.062) 0.241	0.022 (-0.020 to 0.063) 0.303		0.006 (-0.015 to 0.028) 0.563	0.006 (-0.016 to 0.028) 0.585
Mass media expenditure (Immediate impact)			<0.001 (-0.001 to 0.001) 0.873			<0.001 (-0.001 to 0.001) 0.942
	Total percentage change due to the exposure (95%CI) P	Total percentage change due to the exposure (95%CI) P	Total percentage change due to the exposure (95%CI) P	Total percentage change due to the exposure (95%CI) p	Total percentage change due to the exposure (95%CI) P	Total percentage change due to the exposure (95%CI) P
Smoking ban (Pulse effect)			0.017 (-0.069 to 0.103) 0.696			0.014 (-0.071 to 0.099) 0.750
Increase in age-of-sale (Pulse effect)			-0.036 (-0.122 to 0.050) 0.415			-0.040 (-0.125 to 0.044) 0.350
Move to local authority control (Pulse effect)			-0.016 (-0.102 to 0.071) 0.721			-0.026 (-0.111 to 0.060) 0.556
Licensing for NRT for harm reduction (Pulse effect)			0.023 (-0.067 to 0.114) 0.615			0.019 (-0.070 to 0.108) 0.670
NICE guidance on harm reduction (Pulse effect)			-0.021 (-0.106 to 0.065) 0.638			-0.030 (-0.116 to 0.055) 0.483
Best fitting model	ARIMA(0,1,1)(0,0,0)[12]	ARIMA(0,1,1)(0,0,0)[12]	ARIMA(0,1,1)(0,0,0)[12]	ARIMA(0,1,1)(0,0,0)[12]	ARIMA(0,1,1)(0,0,0)[12]	ARIMA(0,1,1)(0,0,0)[12]
Non-seasonal AR *p*	NA	NA	NA	NA	NA	NA
Non-seasonal MA *p*	<0.001	<0.001	<0.001	<0.001	<0.001	<0.001
Seasonal AR *p*	NA	NA	NA	NA	NA	NA
Seasonal MA *p*	NA	NA	NA	NA	NA	NA
R-squared	0.65	0.65	0.65	0.65	0.64	0.65
**Bayes Factor e-cigarette [0.009(0.1)]**	1.01 (0.59)		1.94 (0.38)	1.97 (0.35)		2.15 (0.41)
**Bayes Factor NRT [0.009(0.1)]**		0.15 (0.02)	0.69 (0.11)		1.05 (0.18)	0.61 (0.08)

Note: 95%CI=95% confidence interval; MA=moving average; AR=autoregressive; NA=not applicable; An AR(1) means that the value of a series at one point in time is the sum of a fraction of the value of the series at the immediately preceding point in time and an error component; An MA(1) means that the value of a series at one point in time is a function of a fraction of the error component of the series at the immediately preceding point in time and an error component at the current point in time.

For peer review only - http://bmjopen.bmj.com/site/about/guidelines.xhtml

References

1. Moore D, Aveyard P, Connock M, et al. Effectiveness and safety of nicotine replacement therapy assisted reduction to stop smoking: systematic review and meta-analysis. Bmj 2009;**338**:b1024
2. Beard E, McNeill A, Aveyard P, et al. Use of nicotine replacement therapy for smoking reduction and during enforced temporary abstinence: a national survey of English smokers. Addiction 2011;**106** doi: 10.1111/j.1360-0443.2010.03215.x[published Online First: Epub Date]|.
3. Beard E, Michie S, Fidler J, et al. Use of nicotine replacement therapy in situations involving temporary abstinence from smoking: a national survey of English smokers. Addictive behaviors 2013;**38**(3):1876-79
4. Beard E, Aveyard P, Michie S, et al. Does use of nicotine replacement therapy while continuing to smoke undermine cessation?: a systematic review. Journal of Smoking Cessation 2013;**8**(01):45-56
5. Beard E, Vangeli E, Michie S, et al. The use of nicotine replacement therapy for smoking reduction and temporary abstinence: an interview study. Nicotine & Tobacco Research 2012;**14**(7):849-56
6. Beard E, Bruguera C, McNeill A, et al. Association of amount and duration of NRT use in smokers with cigarette consumption and motivation to stop smoking: A national survey of smokers in England. Addictive behaviors 2015;**40**:33-38
7. Etter JF, Bullen C. Electronic cigarette: users profile, utilization, satisfaction and perceived efficacy. Addiction 2011;**106**(11):2017-28
8. Etter J-F. Electronic cigarettes: a survey of users. BMC public health 2010;**10**(1):1
9. Beard E, Brown J, McNeill A, et al. Has growth in electronic cigarette use by smokers been responsible for the decline in use of licensed nicotine products? Findings from repeated cross-sectional surveys. Thorax 2015:thoraxjnl-2015-206801
10. Vansickel AR, Cobb CO, Weaver MF, et al. A clinical laboratory model for evaluating the acute effects of electronic "cigarettes": nicotine delivery profile and cardiovascular and subjective effects. Cancer Epidemiology Biomarkers & Prevention 2010;**19**(8):1945-53
11. Bullen C, Howe C, Laugesen M, et al. Electronic cigarettes for smoking cessation: a randomised controlled trial. The Lancet 2013;**382**(9905):1629-37
12. Kralikova E, Kubatova S, Truneckova K, et al. The electronic cigarette: what proportion of smokers have tried it and how many use it regularly? Addiction 2012;**107**(8):1528-29
13. Dawkins L. Electronic cigarettes: what are they and are they effective? E-Cigarette Summit, London, UK: (oral presentation). Secondary Electronic cigarettes: what are they and are they effective? E-Cigarette Summit, London, UK: (oral presentation). 2013. http://e-cigarette-summit.com/wp-content/uploads/2013/12/Summit-Presentations.pdf.
14. Polosa R, Caponnetto P, Morjaria JB, et al. Effect of an electronic nicotine delivery device (e-Cigarette) on smoking reduction and cessation: a prospective 6-month pilot study. BMC public health 2011;**11**(1):1
15. McRobbie H, Bullen C, Hartmann-Boyce J, et al. Electronic cigarettes for smoking cessation and reduction. Cochrane Database Syst Rev 2014;**12**
16. Box GE, Jenkins GM, Reinsel GC. *Time series analysis: forecasting and control*: John Wiley & Sons, 2011.
17. Gallus S, Schiaffino A, Vecchia CL, et al. Price and cigarette consumption in Europe. Tobacco Control 2006;**15**(2):114-19 doi: 10.1136/tc.2005.012468[published Online First: Epub Date]|.
18. Langley T, Szatkowski L, Lewis S, et al. The freeze on mass media campaigns in England: a natural experiment of the impact of tobacco control campaigns on quitting behaviour. Addiction 2014;**109**(6):995-1002

For peer review only - http://bmjopen.bmj.com/site/about/guidelines.xhtml

19. Langley TE, Huang Y, McNeill A, et al. Prescribing of smoking cessation medication in England since the introduction of varenicline. Addiction 2011;**106**(7):1319-24 doi: 10.1111/j.1360-0443.2011.03426.x[published Online First: Epub Date]|.
20. Fidler JA, Shahab L, West O, et al. 'The smoking toolkit study': a national study of smoking and smoking cessation in England. BMC Public Health 2011;**11**(1):479
21. Regan AK, Promoff G, Dube SR, et al. Electronic nicotine delivery systems: adult use and awareness of the ‘e-cigarette’in the USA. Tobacco control 2013;**22**(1):19-23
22. Cho JH, Shin E, Moon S-S. Electronic-cigarette smoking experience among adolescents. Journal of Adolescent Health 2011;**49**(5):542-46
23. Wakefield MA, Durkin S, Spittal MJ, et al. Impact of tobacco control policies and mass media campaigns on monthly adult smoking prevalence. American Journal of Public Health 2008;**98**(8):1443-50
24. Health and Social Care Information Centre. NHS Stop Smoking Services Collection. Secondary NHS Stop Smoking Services Collection 2015. www.hscic.gov.uk/stopsmoking.
25. Hackshaw L, McEwen A, West R, et al. Quit attempts in response to smoke-free legislation in England. Tobacco control 2010;**19**(2):160-64
26. Beard E, Bruguera C, Brown J, et al. Was the Expansion of the Marketing License for Nicotine Replacement Therapy in the United Kingdom to Include Smoking Reduction Associated With Changes in Use and Incidence of Quit Attempts? Nicotine & Tobacco Research 2013;**15**(10):1777-81 doi: 10.1093/ntr/ntt044[published Online First: Epub Date]|.
27. NICE. NICE guidelines [PH45]: Smoking: Harm reduction. Secondary NICE guidelines [PH45]: Smoking: Harm reduction 2013. https://www.nice.org.uk/guidance/ph45.
28. Fidler JA, West R. Changes in smoking prevalence in 16–17-year-old versus older adults following a rise in legal age of sale: findings from an English population study. Addiction 2010;**105**(11):1984-88
29. R Development Core Team. R: A language and environment for statistical computing. R foundation for statistical computing, Vienna, Austria. Secondary R: A language and environment for statistical computing. R foundation for statistical computing, Vienna, Austria. 2008. http://www.R-project.org.
30. Wakefield MA, Coomber K, Durkin SJ, et al. Time series analysis of the impact of tobacco control policies on smoking prevalence among Australian adults, 2001? 2011. Bulletin of the World Health Organization 2014;**92**(6):413-22
31. Cryer JD, Chan K-S. *Time series analysis - with applications in R*. London: Springer-Verlag New York, 2008.
32. Yalta AT. Analyzing energy consumption and GDP nexus using maximum entropy bootstrap: The case of Turkey. Energy Economics 2011;**33**(3):453-60
33. Granger CW. Some recent development in a concept of causality. Journal of econometrics 1988;**39**(1):199-211
34. Box GE, Tiao GC. Intervention analysis with applications to economic and environmental problems. Journal of the American Statistical association 1975;**70**(349):70-79
35. Dienes Z. Using Bayes to get the most out of non-significant results. Frontiers in psychology 2014;**5**
36. Von Elm E, Altman DG, Egger M, et al. The Strengthening the Reporting of Observational Studies in Epidemiology (STROBE) statement: guidelines for reporting observational studies. Preventive medicine 2007;**45**(4):247-51
37. Beard E, Brown J, Michie S, et al. Association between population changes in use of electronic cigarettes and changes in quit attempts, use of smoking cessation pharmacotherapy and use of stop smoking services: a time-series analysis. Under-prepartion
38. Kuipers MAG, Beard E, Hitchman SC, et al. Impact on smoking of England's 2012 partial tobacco point of sale display ban: a repeated cross-sectional national study. Tobacco Control 2016 doi: 10.1136/tobaccocontrol-2015-052724[published Online First: Epub Date]|.

For peer review only - http://bmjopen.bmj.com/site/about/guidelines.xhtml

39. Hitchman SC, Brose LS, Brown J, et al. Associations between e-cigarette type, frequency of use, and quitting smoking: findings from a longitudinal online panel survey in Great Britain. Nicotine & Tobacco Research 2015:ntv078
40. Little RJ, Rubin DB. *Statistical analysis with missing data*: John Wiley & Sons, 2014.
41. Shi Y, Pierce JP, White M, et al. E-cigarette use and smoking reduction or cessation in the 2010/2011 TUS-CPS longitudinal cohort. BMC Public Health 2016;**16**(1):1105 doi: 10.1186/s12889-016-3770-x[published Online First: Epub Date]|.
42. Brose LS, Hitchman SC, Brown J, et al. Is the use of electronic cigarettes while smoking associated with smoking cessation attempts, cessation and reduced cigarette consumption? A survey with a 1-year follow-up. Addiction 2015;**110**(7):1160-8 doi: 10.1111/add.12917[published Online First: Epub Date]|.

For peer review only

For peer review only - http://bmjopen.bmj.com/site/about/guidelines.xhtml

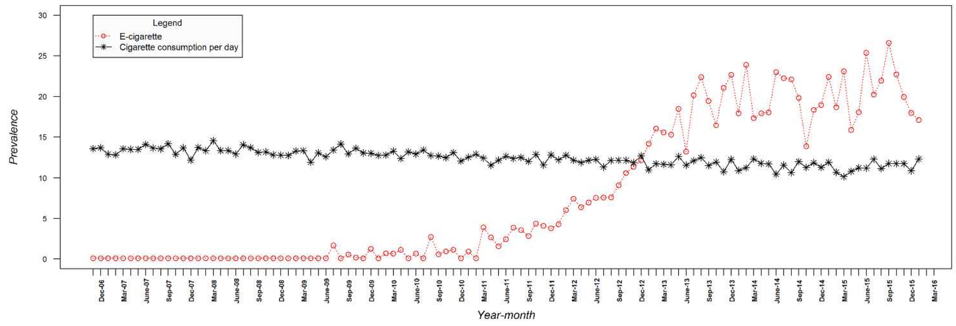

Figure 1: Monthly prevalence of cigarette consumption and e-cigarette use for harm reduction among smokers

381x177mm (300 x 300 DPI)

For peer review only

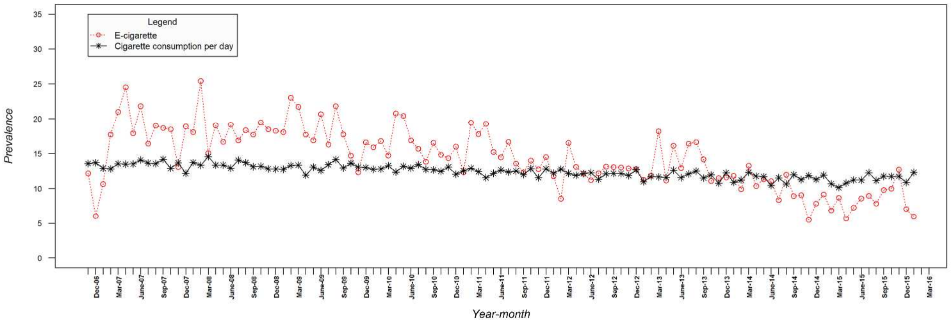

Figure 2: Monthly prevalence of cigarette consumption and NRT use for harm reduction among smokers

381x177mm (300 x 300 DPI)

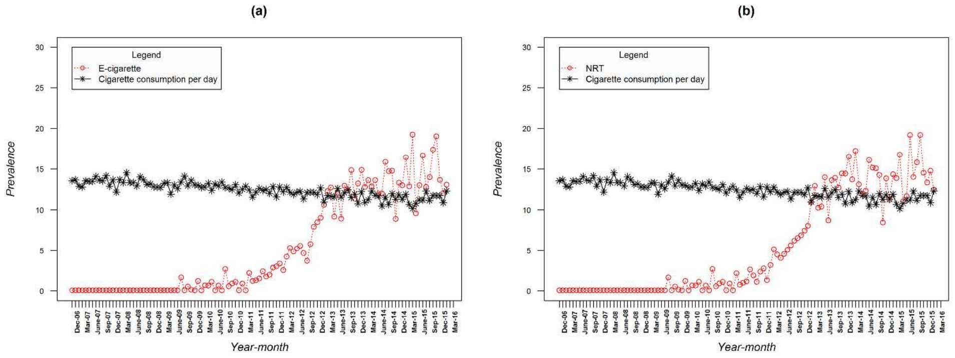

Supplementary Figure 1: Monthly prevalence of cigarette consumption and e-cigarette use for a) cutting down and b) temporary abstinence among smokers

381x177mm (300 x 300 DPI)

For peer review only

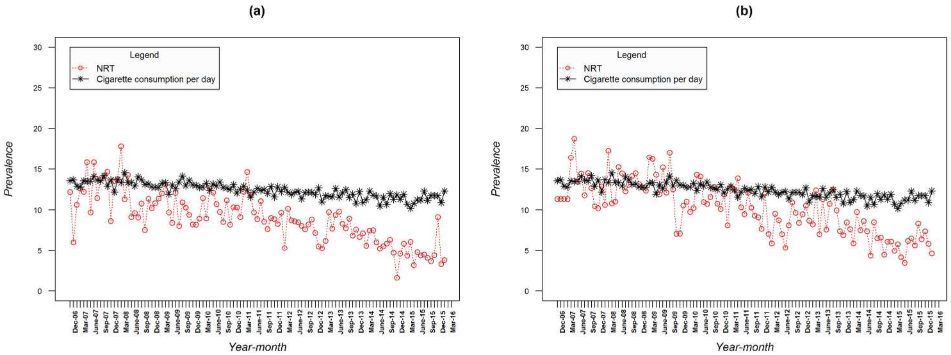

Supplementary Figure 2: Monthly prevalence of cigarette consumption and NRT use for a) cutting down and b) temporary abstinence among smokers

381x177mm (300 x 300 DPI)

For peer review only

STROBE 2007 (v4) Statement—Checklist of items that should be included in reports of *cross-sectional studies*

Section/Topic	Item #	Recommendation	Reported on page #
**Title and abstract**	1	(*a*) Indicate the study's design with a commonly used term in the title or the abstract	1
		(*b*) Provide in the abstract an informative and balanced summary of what was done and what was found	2
**Introduction**			
Background/rationale	2	Explain the scientific background and rationale for the investigation being reported	3
Objectives	3	State specific objectives, including any prespecified hypotheses	3
**Methods**			
Study design	4	Present key elements of study design early in the paper	4/5
Setting	5	Describe the setting, locations, and relevant dates, including periods of recruitment, exposure, follow-up, and data collection	4/5
Participants	6	(*a*) Give the eligibility criteria, and the sources and methods of selection of participants	4/5
Variables	7	Clearly define all outcomes, exposures, predictors, potential confounders, and effect modifiers. Give diagnostic criteria, if applicable	4/5
Data sources/ measurement	8*	For each variable of interest, give sources of data and details of methods of assessment (measurement). Describe comparability of assessment methods if there is more than one group	4/5
Bias	9	Describe any efforts to address potential sources of bias	*5/6*
Study size	10	Explain how the study size was arrived at	6
Quantitative variables	11	Explain how quantitative variables were handled in the analyses. If applicable, describe which groupings were chosen and why	5/6
Statistical methods	12	(*a*) Describe all statistical methods, including those used to control for confounding	5/6
		(*b*) Describe any methods used to examine subgroups and interactions	5/6
		(*c*) Explain how missing data were addressed	5/6
		(*d*) If applicable, describe analytical methods taking account of sampling strategy	5./6
		(*e*) Describe any sensitivity analyses	5/6
**Results**			

For peer review only

Participants	13*	(a) Report numbers of individuals at each stage of study—eg numbers potentially eligible, examined for eligibility, confirmed eligible, included in the study, completing follow-up, and analysed	6
		(b) Give reasons for non-participation at each stage	N/A
N/A		(c) Consider use of a flow diagram	
Descriptive data	14*	(a) Give characteristics of study participants (eg demographic, clinical, social) and information on exposures and potential confounders	6
		(b) Indicate number of participants with missing data for each variable of interest	N/A
Outcome data	15*	Report numbers of outcome events or summary measures	6/7
Main results	16	(*a*) Give unadjusted estimates and, if applicable, confounder-adjusted estimates and their precision (eg, 95% confidence interval). Make clear which confounders were adjusted for and why they were included	6/7
		(*b*) Report category boundaries when continuous variables were categorized	N/A
		(*c*) If relevant, consider translating estimates of relative risk into absolute risk for a meaningful time period	N/A
Other analyses	17	Report other analyses done—eg analyses of subgroups and interactions, and sensitivity analyses	6/7
**Discussion**			
Key results	18	Summarise key results with reference to study objectives	7/8
Limitations	19	Discuss limitations of the study, taking into account sources of potential bias or imprecision. Discuss both direction and magnitude of any potential bias	7/8
Interpretation	20	Give a cautious overall interpretation of results considering objectives, limitations, multiplicity of analyses, results from similar studies, and other relevant evidence	7/8
Generalisability	21	Discuss the generalisability (external validity) of the study results	7/8
**Other information**			
Funding	22	Give the source of funding and the role of the funders for the present study and, if applicable, for the original study on which the present article is based	8

*Give information separately for cases and controls in case-control studies and, if applicable, for exposed and unexposed groups in cohort and cross-sectional studies.

Note: An Explanation and Elaboration article discusses each checklist item and gives methodological background and published examples of transparent reporting. The STROBE checklist is best used in conjunction with this article (freely available on the Web sites of PLoS Medicine at http://www.plosmedicine.org/, Annals of Internal Medicine at http://www.annals.org/, and Epidemiology at http://www.epidem.com/). Information on the STROBE Initiative is available at www.strobe-statement.org.

For peer review only
